# Persistently Elevated Gamma Power and Delayed Brain Damage in Aged Rats Acutely Exposed to Soman Without Status Epilepticus: Comparisons with Seizing Rats Treated with Midazolam or with Tezampanel and Caramiphen

**DOI:** 10.3390/toxics14010022

**Published:** 2025-12-25

**Authors:** Taiza H. Figueiredo, Vassiliki Aroniadou-Anderjaska, Marcio De Araujo Furtado, Volodymyr I. Pidoplichko, Katia Rossetti, Lucille A. Lumley, Maria F. M. Braga

**Affiliations:** 1 Department of Anatomy, Physiology, and Genetics, F. Edward Hébert School of Medicine, Uniformed Services University of the Health Sciences, Bethesda, MD 20814, USA; taiza.figueiredo.ctr@usuhs.edu (T.H.F.); vanderjaska@usuhs.edu (V.A.-A.); mdafurtado@gmail.com (M.D.A.F.); volodymyr.pidoplichko.ctr@usuhs.edu (V.I.P.); katia.rossetti.ctr@usuhs.edu (K.R.); 2 Department of Psychiatry, F. Edward Hébert School of Medicine, Uniformed Services University of the Health Sciences, Bethesda, MD 20814, USA; 3 U.S. Army Medical Research Institute of Chemical Defense, Aberdeen Proving Ground, Aberdeen, MD 21010, USA

**Keywords:** status epilepticus, tezampanel, midazolam, caramiphen, nerve agents, organophosphates, gamma band, amygdala

## Abstract

Aged animals or humans are more susceptible to permanent brain damage from status epilepticus (SE), making the selection of antiseizure medication even more crucial. This study compared the antiseizure and neuroprotective efficacy of midazolam with that of tezampanel combined with caramiphen in treating soman-induced SE in aged rats. A substantial proportion of soman-exposed aged rats did not develop SE, allowing us to also study this noSE group. SE duration within 24 h post-exposure was significantly longer in the midazolam than the tezampanel + caramiphen group, which was reflected in the EEG power integral. Spectral density analysis showed sustained increase in gamma-band power in the noSE group. Increased delta power in the SE groups lasted longer after midazolam. Body temperature decreased substantially only in the noSE and tezampanel + caramiphen groups. The midazolam group displayed severe neuropathology in the hippocampus and the amygdala 7 days to 6 months post-exposure, whereas the noSE and tezampanel + caramiphen groups exhibited only delayed amygdala damage. Thus, tezampanel + caramiphen has far superior neuroprotective efficacy than midazolam in aged rats. Increased gamma power is associated with seizure resistance; however, even in the absence of SE, delayed neuropathology can develop after a single acute organophosphate exposure.

## 1. Introduction

The elderly are more susceptible to mortality or permanent brain injury that can be caused by an episode of status epilepticus (SE) [1,2,3]. In addition, the incidence of SE is higher in the older population [1,3], and the aging demographic is expanding [4]. These considerations underscore the critical importance of the antiseizure and neuroprotective effectiveness of medications used to control SE in older patients.

Status epilepticus in older adults can be triggered by various causes [1], including exposure to certain toxins. Organophosphorus compounds (OPs)—a class that includes insecticides as well as nerve agents [5,6]—can be highly toxic, depending on the type, dose, and duration of exposure. Acute exposure to high concentrations of OPs, which may occur accidentally or, in the case of nerve agents, as a result of a terrorist attack, can cause SE. The mechanism of SE induction by OPs involves phosphorylation of the catalytic site of acetylcholinesterase [7]—the enzyme that hydrolyzes acetylcholine—which inactivates the enzyme; the result is excessive elevation of acetylcholine in cholinergic synapses and hyperstimulation of nicotinic and muscarinic cholinergic receptors in the peripheral and central nervous system [5,6].

In the central nervous system, initiation of SE is primarily due to overstimulation of muscarinic receptors; however, within a few minutes from SE onset, glutamatergic hyperactivity takes over, strengthening and sustaining seizures [7]. Because glutamatergic hyperexcitation can be suppressed by enhancing inhibitory neurotransmission, the FDA has approved the use of benzodiazepines (BZs)—which increase inhibition by enhancing the activity of GABA_A_ receptors [8]—for the treatment of OP/nerve agent-induced SE. The approval was initially for diazepam, which was recently replaced by midazolam (MDZ) [9,10], primarily due to its water solubility and extended shelf life at room temperature [10,11]. However, only when diazepam or MDZ are administered promptly after SE onset (within ~15 min) can seizures be suppressed effectively [12,13,14,15,16], while delayed BZ treatment is associated with significant seizure reoccurrence [17,18,19] and brain damage [15,16,17,19,20,21,22,23]. The decrease in BZ antiseizure and neuroprotective efficacy as the latency from SE onset increases is mainly due to internalization of synaptic GABA_A_ receptors as SE progresses [24,25]; other possible mechanisms include disruption of chloride homeostasis or further decrease in GABA_A_ receptor availability by sustained BZ exposure [26,27].

Many patients experience delays of more than 30 min before receiving first-line treatment for SE [28]—delays that almost certainly would be longer in a mass-exposure scenario involving an SE-inducing agent. Given this, it is remarkable that due emphasis has not been placed on replacing BZs as first-line therapy with novel, more effective antiseizure medications. Such medications could directly target hyperexcitation by antagonizing glutamate receptors, which—unlike synaptic GABA_A_ receptors that are internalized during SE—are upregulated [24,29,30,31,32].

We have previously compared the antiseizure and neuroprotective efficacy of diazepam or MDZ, in immature and young-adult rats exposed to the nerve agent soman, with that of tezampanel (LY293558, an antagonist of AMPA receptors and kainate receptors containing the GluK1 subunit [33]) administered alone or in combination with caramiphen (CRM) [18,19,23,34,35], which is an antimuscarinic compound with NMDA receptor antagonistic properties [36,37]. These studies showed a far superior neuroprotective efficacy of the antiglutamatergic therapy, which was enhanced with the addition of CRM [34,38]. Antiseizure medications that are effective in the young population are not necessarily equally effective in older adults [39,40,41], who are more vulnerable to mortality and brain damage from SE [1,2,3]. Therefore, in the present study, we compared the antiseizure and long-term neuroprotective efficacy of MDZ with that of LY293558 administered along with CRM in aged male rats exposed to soman. In addition, because delayed brain damage has been reported after acute OP exposure in the absence of SE [42], we also studied those rats that did not develop SE after soman exposure.

## 2. Materials and Methods

### 2.1. Animals

Ten-month-old, male Sprague Dawley rats (500–790 g) were purchased from Charles River Laboratories (Wilmington, MA, USA). The rats were individually housed in an environmentally controlled room (20–23 °C, 12 h light/12 h dark cycle, lights on 06:00 am), with food and water available ad libitum. The experiments followed the Guide for the Care and Use of Laboratory Animals (Institute of Laboratory Animal Resources, National Research Council) and were approved by the Institutional Animal Care and Use Committees of the Uniformed Services University of the Health Sciences (USUHS) and the U.S. Army Medical Research Institute of Chemical Defense (MRICD). The animal care and use programs of both institutions are accredited by the Association for Assessment and Accreditation of Laboratory Animal Care International.

### 2.2. Soman Administration and Drug Treatment

Exposure to soman took place at the MRICD. The rats were subcutaneously (s.c.) injected with soman (pinacolyl methylphosphonofluoridate; obtained from the United States Army Combat Capabilities Development Command Chemical Biological Center, Aberdeen Proving Ground, Gunpowder, MD, USA) at the dose of 71 μg/kg (1.2 × LD_50_, [43]); this was followed by atropine sulfate [2 mg/kg, intramuscularly (i.m.); Sigma-Aldrich, St. Louis, MO, USA] and HI-6 [125 mg/kg, intraperitoneally (i.p.); Starks Associates, Buffalo, NY, USA], administered within 1 min after soman injection to control the peripheral cholinergic crisis. Sixty percent of the rats did not develop SE after the initial soman injection. To increase the proportion of rats that developed SE, we administered one or two additional low doses of soman (at 1/4 of the initial dose, 17.75 μg/kg) to some of those rats that had not developed SE by 20 min after soman injection (for specific numbers of rats see 1st paragraph of Section 3 and Table 1). This procedure facilitated seizure induction; however, a few rats still did not develop SE despite administration of two additional low soman doses. Rats that did not develop SE constituted the “noSE group”. The rats that developed SE (“SE rats”), either after a single soman injection or following one or two additional low-dose injections, received MDZ (5 mg/kg, i.m.; Hospira Inc., Lake Forest, IL, USA) or LY293558 (10 mg/kg, i.m.; MedKoo Biosciences Inc., Morrisville, NC, USA) along with CRM (50 mg/kg, i.m.; Sigma-Aldrich, St Louis, MO, USA) at 30 min after SE onset. The doses for each anticonvulsant have been determined in previous studies [19,37] and have been adjusted so as to stop seizures within about 30 min after administration, without causing cardiorespiratory depression. To reduce mortality before administration of the anticonvulsants, all SE rats received an additional dose of atropine (2 mg/kg, i.m.) 15 min after the first stage 3 behavioral seizure, which indicates the onset of SE. The occurrence of convulsive SE was determined by behavioral observations based on the Racine scale [44], as described previously [37,45]; a subset of rats was implanted with electrodes for electroencephalographic (EEG) recordings (see Section 2.3).

Following soman exposure, the rats were returned to USUHS, frequently monitored, and administered Lactated Ringer’s, 5 mL (s.c.), up to 3 times a day for 2 days or longer, until body weight stabilized. The rats also received Electro-gel cups (Bio Serv Inc., Farmington, NJ, USA) for 2 days and moistened chow for up to 10 days.

### 2.3. Electrode Implantation and EEG Recordings

Rats were implanted with subcutaneous electroencephalographic (EEG) telemetry electrodes, as described in Lumley et al. [46]. Prior to surgery, the rats received meloxicam (1 mg/kg, s.c.; Patterson Veterinary, St. Paul, MN, USA), a non-steroidal anti-inflammatory drug. Under anesthesia with isoflurane (3–5% induction, 1.5–5% maintenance), the rats were positioned securely in a Kopf stereotaxic apparatus (David Kopf Instruments, Tujunga, CA, USA). Two cortical stainless steel screw electrodes were placed in the skull, 2 mm to the left of the midline, at 1.6 mm anterior and 4 mm posterior to bregma. ETA-F10 transmitters with stainless steel wires (Data Sciences International Inc., St. Paul, MN, USA) were implanted subcutaneously, with the wires wrapped around the electrodes and secured with dental acrylic. After surgery, rats were removed from anesthesia and administered buprenorphine sustained release (1.0 mg/kg, s.c.; ZooPharm, Laramie, WY, USA). They were then given 7–10 days to recover before transported to MRICD for soman exposure.

EEG was recorded continuously using Ponemah software v6.51 (DSI Inc., St. Paul, MN, USA) with an RPC-1 PhysioTel receiver (DSI Inc.) placed under each animal’s cage. Recordings began at least 1 h before soman exposure and continued until 24 h post-exposure. EEG data were digitized at 250 Hz. Seizure onset and acute seizure duration were determined through visual screening by a blinded and unbiased scorer using a customized MATLAB graphical user interface (MATLAB R2018b; The MathWorks, Inc., Natick, MA, USA). The cessation of SE was defined as the disappearance of large-amplitude, repetitive discharges (>1 Hz with at least double the amplitude of background activity). The EEG power spectrum was calculated using a customized MATLAB algorithm (MATLAB R2018b; The MathWorks, Inc., Natick, MA, USA [47]) and divided in the following bands: delta (0.1–4.0 Hz), theta (4.1–8.0 Hz), alpha (8.1–12 Hz), beta (12.1–25 Hz), and low gamma (25.1–50 Hz). The power integral was calculated by determining the power spectra (μV2/Hz; 0.1–125 Hz) of each period through a customized MATLAB algorithm and applying the formula [48]:

[decibels = 10 × (Log(V^2^sample/V^2^normal))] × epoch duration

EEG power spectrum data were additionally reduced by extracting the median power in 10 and 60 min intervals to obtain EEG power spectral density values at baseline (1 h prior to soman exposure) and up to 24 h after exposure.

### 2.4. Tissue Processing

Rats were deeply anesthetized with pentobarbital (75–100 mg/kg, i.p.) and transcardially perfused with PBS (100 mL), followed by 4% paraformaldehyde (200 mL). The brains were removed and post-fixed in 4% paraformaldehyde overnight at 4 °C. The following day, the brains were transferred to a solution of 30% sucrose in PBS for 72 h, then frozen using slow immersion in 2-methylbutane surrounded by dry-ice and stored at −20 °C until sectioning. Sections were cut at a thickness of 40 µm using a sliding microtome, and a 1-in-5 series (one out of every five sections) was collected, spanning from the rostral extent of the amygdala to the caudal extent of the entorhinal cortex. One series of sections was mounted on slides (Superfrost Plus, Daigger, Vernon Hills, IL, USA) in PBS for Nissl staining with cresyl violet; these sections were used for total neuronal counting and estimation of neuronal loss. Adjacent series were mounted on slides for Fluoro-Jade C (FJC) staining (used to determine the extent of neurodegeneration), or were stored at −20 °C in cryoprotectant solution for glutamic acid decarboxylase-67 (GAD-67) immunohistochemical labeling (used for identification and counting of interneurons). All neuropathological analyses were conducted in a blinded manner.

### 2.5. Fluoro-Jade C Staining and Analysis

Neurodegeneration was assessed in the amygdala, piriform cortex, entorhinal cortex, a sample of temporal neocortex, and the CA1, CA3, and hilar areas of the ventral hippocampus, as the ventral hippocampus exhibits more severe neurodegeneration after soman exposure compared with the dorsal hippocampus [49]. The procedure for staining irreversibly degenerating neurons with FJC (Histo-Chem, Jefferson, AR, USA) was performed as described previously [17,44]. FJC-stained sections were superimposed onto tracings of regions of interest from adjacent Nissl-stained sections using Stereo Investigator 2017 (MicroBrightField, Williston, VT, USA). Neurodegeneration was quantified by comparing the density of FJC-stained cells to that of Nissl-stained cells across the anterior–posterior axis at 600 μm intervals. Damage severity in each structure was scored on a scale of 0 to 4 (0 = no damage, 1 = minimal [1–10%], 2 = mild [11–25%], 3 = moderate [26–45%], 4 = severe [>45%]), based on qualitative assessments of 6 sections per animal, with the average recorded for each animal.

### 2.6. GAD-67 Immunolabeling

Sections were removed from the cryoprotectant solution and washed three times for 5 min each in 0.1 M PBS. They were then incubated for 1 h at room temperature in a blocking solution containing 10% normal goat serum (MilliporeSigma, Temecula, CA, USA) and 0.5% Triton X-100 in PBS. Next, the sections were incubated overnight at 4 °C with a primary antibody solution consisting of mouse anti-GAD-67 serum (1:1000, MAB5406; Chemicon, Darmstadt, Germany), 5% normal goat serum, 0.3% Triton X-100, and 1% bovine serum albumin in PBS. After three washes of 10 min each in 0.1% Triton X-100 in PBS, the sections were incubated for 1 h at room temperature with Cy3-conjugated goat anti-mouse secondary antibody (1:1000; Jackson ImmunoResearch, West Grove, PA, USA) and 0.0001% DAPI (Sigma, St. Louis, MO, USA) in PBS. Following the incubation, the sections were rinsed in PBS for 10 min, mounted on slides, air-dried for 30 min, and coverslipped using ProLong Gold antifade reagent (Invitrogen, Waltham, MA, USA).

### 2.7. Estimation of Neuronal and Interneuronal Loss

Design-based stereology was used to quantify the total number of neurons in Nissl-stained sections and interneurons in GAD-67-immunostained sections in the CA1 hippocampal area and the basolateral amygdala (BLA). We focused the analysis of neuron and interneuron loss, as well as the volumetric analysis (described in the next section), on the hippocampus and the amygdala because these limbic structures play central roles in seizure generation and propagation [50,51] and are key to cognitive functions and emotional behavior [52,53,54].

Sections were examined using a Zeiss Axioplan 2ie fluorescent microscope (Oberkochen, Germany) equipped with a motorized stage and interfaced with a computer running Stereo Investigator 2017 (MicroBrightField, Williston, VT, USA). Tracings of the CA1 and the BLA were made on both hemispheres (bilaterally) under a 2.5× objective, and counting was performed under a 63× oil immersion objective. The optical fractionator probe was used to estimate the total number of Nissl-stained and GAD-67-immunostained neurons, and the coefficient of error (CE) was calculated using Stereo Investigator 2017. The CE was determined according to Gundersen (m = 1) and Schmitz-Hof (2nd estimation) equations [55,56].

To determine the number of Nissl-stained neurons in the CA1 area, 1 section in a series of 10 sections was analyzed, with an average of 6 sections per rat. Bilateral tracings of CA1 were made under 2.5× objective. As tissue damage can make the boundary between the CA1 and CA2 regions difficult to define, contributions from CA2 may also be present in the counts. The counting frame was 20 μm × 20 µm, the counting grid was 250 μm × 250 µm, and the dissector height was 10 µm. Nuclei were counted when the cell body came into focus within the dissector, placed 2 µm below the section surface. Section thickness was measured at each counting site, and the average mounted section thickness was 19.1 µm. An average of 245 neurons per rat was counted, with a CE of 0.02 for Gundersen (m = 1) and 0.03 for the Schmitz-Hof (2nd estimation) equation.

To determine the number of Nissl-stained neurons in the BLA, 1 section in a series of 5 sections was analyzed, with an average of 6 sections per rat. The counting frame was 35 μm × 35 µm, the counting grid was 190 μm × 190 µm, and the dissector height was 12 µm. Nuclei were counted when the cell body came into focus within the dissector, which was placed 2 µm below the section surface. Section thickness was measured at every counting site, and the average mounted section thickness was 20.1 µm. On average, 291 neurons per rat were counted, with a CE of 0.03 for Gundersen (m = 1) and 0.04 for Schmitz-Hof (2nd estimation) equations.

To determine the number of GAD-67-immunolabeled neurons in the CA1 area and the BLA, 1 section in a series of 10 sections was analyzed, with an average of 5 sections per rat. The counting frame was 60 μm × 60 µm, the counting grid was 100 μm × 100 µm, and the dissector height was 20 µm. Nuclei were counted when the top of the nucleus came into focus within the dissector, placed 2 µm below the section surface. Section thickness was measured at every 5th counting site, and the average mounted section thickness was 30 µm. On average, 220 neurons per rat were counted, with a CE of 0.05 for the Gundersen equation and 0.06 for Schmitz-Hof equation.

### 2.8. Volumetric Analysis

Nissl-stained sections containing the hippocampus (spaced 400 µm apart) or the amygdala (spaced 200 µm apart) were used to stereologically estimate the volume of these structures using the Cavalieri principle [57]. Sections were viewed with a Zeiss Axioplan 2ie fluorescent microscope (Oberkochen, Germany) equipped with a motorized stage and interfaced with a computer running Stereo Investigator 2017. The amygdala and hippocampus were identified on slide-mounted sections under a 2.5× objective, based on the atlas of Paxinos and Watson [58], and traced using Stereo Investigator 2017. The coordinates for the amygdala were from bregma −2.6 to bregma −3.6, and for the hippocampus, from bregma −2.3 to bregma −6.3. Volumes were calculated using the Cavalieri Estimator probe. A rectangular lattice grid with a spacing of 300 µm was overlaid on the tracings, and the points marked were counted to estimate the volume. For each animal, the CE was calculated to ensure sufficient accuracy (CE < 0.05).

### 2.9. Electrophysiological Experiments

Whole-cell recordings of spontaneous IPSCs (sIPSCs) were obtained from the BLA. The experiments were conducted in a blind fashion; the electrophysiologist was not informed of the anticonvulsant treatment the rat had received or of whether the rat belonged to the noSE group. Rats were anesthetized with isoflurane before decapitation. Coronal brain slices (400 µm thick) containing the amygdala were cut in ice-cold solution containing (in mM): 115 sucrose, 70 NMDG, 1 KCl, 2 CaCl_2_, 4 MgCl_2_, 1.25 NaH_2_PO_4_, 30 NaHCO_3_, using a vibratome (Leica VT 1200 S; Leica Microsystems, Buffalo Grove, IL, USA). Slices were transferred to a holding chamber at room temperature in a bath solution containing (in mM): 125 NaCl, 2.5 KCl, 1.25 NaH_2_PO_4,_ 21 NaHCO_3_, 2 CaCl_2_, 1 MgCl_2_, and 10 D-glucose. The recording solution (artificial cerebrospinal fluid; ACSF) was identical to the holding bath solution. All solutions were saturated with 95% O_2_/5% CO_2_ to maintain a pH near 7.4. The recording chamber (0.7 mL capacity) was perfused continuously with ACSF (~8 mL/min) at 30–31 °C. The osmolarities of both the cutting solution and the ACSF were adjusted to 325 mOsm with D-glucose.

To visualize neurons in the BLA, we used a 40× water immersion objective equipped with a CCD-100 camera (Dage-MTI, Michigan City, IN, USA), under infrared light and Nomarski optics of an upright microscope (Zeiss Axioskop 2, Thornwood, NY, USA). Recording electrodes had resistances of 3.5–4.5 MΩ when filled with the internal solution containing (in mM): 60 CsCH_3_SO_3_, 60 KCH_3_SO_3_, 5 KCl, 10 EGTA, 10 HEPES, 5 Mg-ATP, and 0.3 Na_3_GTP (pH 7.2; osmolarity adjusted to 295 mOsm with potassium gluconate). Tight-seal (over 1 GΩ) whole-cell recordings were obtained from the cell bodies of principal neurons, distinguished from interneurons by their larger size, pyramidal shape, and electrophysiological characteristics [59,60,61,62]. Access resistance (under 20 MΩ) was regularly monitored during recordings, and cells were excluded if resistance changed by more than 15% during the experiment. Currents were amplified and filtered using an Axopatch 200B amplifier (Axon Instruments, Foster City, CA, USA) with a four-pole, low-pass Bessel filter. Signals were digitally sampled (up to 2 kHz) using Clampex 10.7 software (Molecular Devices, Sunnyvale, CA, USA) and subsequently analyzed using Origin 2019b software (OriginLab Corporation, Northampton, MA, USA).

### 2.10. Statistical Analysis

Statistical comparisons were made between the SE rats treated with MDZ or LY293558 + CRM, and with age-matched control groups (not exposed to soman) where appropriate. The noSE rats were compared with either control groups or baseline values, depending on the variable. Differences in 24 h survival rate between soman-exposed groups treated with MDZ or LY293558 + CRM were tested for statistical significance using Fisher’s exact test. Long-term survival differences between groups were assessed by the Kaplan–Meier method and the log-rank (Mantel–Cox) test. Comparisons between the two treatments in the latency to cessation of the initial SE (defined as SE up to its transient cessation by anticonvulsant treatment), duration of the initial SE, and total duration of SE in 24 h post-exposure were made using Welch’s *t*-test (unequal variances *t*-test). Within-group comparisons of EEG Power Integral values versus baseline (zero) were performed using one-sample *t*-tests. For body temperature and EEG power spectrum, comparisons to baseline at each timepoint were conducted using paired *t*-tests. Between-group differences in EEG Power Integral, body temperature, and EEG power spectrum were assessed with Student’s *t*-tests.

Differences between groups in neuronal degeneration were tested for significance with the use of the Mann–Whitney U-test. Neuronal and interneuronal loss in the CA1 hippocampal area and the BLA was compared between experimental groups and controls using ANOVA with LSD post hoc test. The volumes of the hippocampus and the amygdala were compared between experimental groups and controls using one-way ANOVA followed by Bonferroni post hoc tests. The percentage of BLA neurons exhibiting sIPSC “bursts” was compared between experimental groups and controls using Fisher’s exact test, while Mann–Whitney U-tests were used to analyze differences in charge transfer and frequency of sIPSC bursts.

The statistical tests used are stated in each figure legend. Statistical values are presented as means ± standard errors of the mean, except for neurodegeneration scores, which are reported as medians and interquartile ranges (IQR, the difference between the 75th and the 25th percentiles). For all tests, differences were considered statistically significant at *p* < 0.05. Sample sizes (*n*) refer to the number of animals, except for the in vitro experiments, where *n* refers to the number of recorded neurons, which were obtained from 3 to 4 rats per group per timepoint.

## 3. Results

A total of 119 aged male rats were exposed to soman, followed by atropine and HI-6. Forty-six of these rats developed SE within 13.23 ± 1.4 min (*n* = 46). However, 73 rats (about 60%) did not develop SE by 20 min after soman injection. Of these, 16 were kept for the formation of the noSE group, while the remaining 57 rats were administered one or two additional low-dose injections of soman (see Methods) in order to increase the number of rats that developed SE. From these 57 rats, 11 did not develop SE despite administration of two additional low-dose soman injections; these 11 rats were added to the noSE group, resulting in 27 rats in this group (23% of all exposed rats). None of the noSE rats developed SE at later timepoints within the 24 h post-exposure period. Of the 92 rats that developed SE (46 after one soman injection and 46 after one or two additional low-dose soman injections), one died before anticonvulsant treatment, 41 were treated with MDZ, and 50 with LY293558 + CRM. Of the rats that received one or two additional low doses of soman, 22 and 23 were randomly assigned to the MDZ and LY293558 + CRM groups, respectively. For both treatment groups, the 24 h survival rate was 90% (Table 1).

**Table 1:** Number of rats exposed to soman and 24 h survival in SE rats treated with MDZ (5 mg/kg) or LY293558 (10 mg/kg) along with CRM (50 mg/kg) at 30 min after SE onset.

Number of Aged Male Rats Exposed to Soman	Did Not Develop SE	Died Before Anticonvulsant Treatment	Received Anticonvulsant Treatment	24 h Survival Rate
119	27 (23%)	1	41 MDZ	90% (37/41)
			50 LY293558 + CRM	90% (45/50)

**Figure 1:**
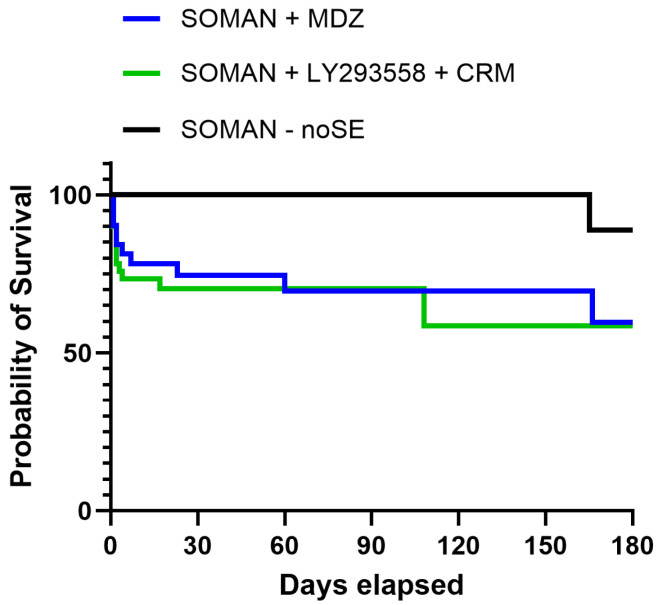
Kaplan–Meier survival curve for a period of 6 months after exposure of aged male rats to soman, followed by treatment with MDZ or LY293558 + CRM. The difference in probability of survival between the two SE groups, one treated with MDZ and the other with LY293558 + CRM, was not statistically significant. Survival distributions were compared using the log-rank (Mantel–Cox) test.

The probability of survival over time was somewhat lower in the LY293558 + CRM group than in the MDZ group, but the difference was not statistically significant (*p* = 0.8, Mantel–Cox test; Figure 1). In the MDZ group, two rats died on day 2, and a single rat death occurred on days 4, 7, 23, 60, and 166. In the LY293558 + CRM group, three rats died on day 2, and a single rat death occurred on days 3, 4, 17, and 108. In the noSE group, one rat died on day 165.

### 3.1. Shorter Duration of SE in Rats Treated with LY293558 + CRM Compared with MDZ

**Figure 2:**
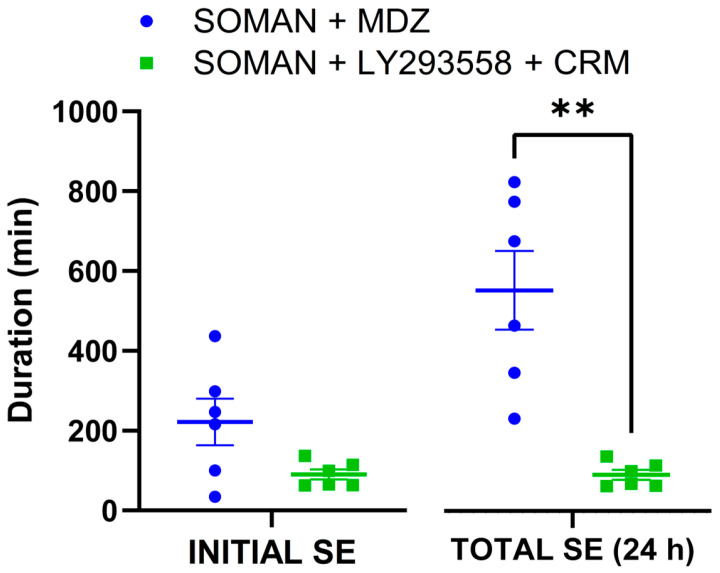
Scatter plots showing means, standard errors, and individual data points for initial SE and total duration of SE within 24 h after soman exposure in aged male rats treated with MDZ or LY293558 + CRM 30 min after SE onset. There was substantially greater variability in the duration of SE in response to MDZ treatment. The total duration of SE was significantly longer in the MDZ group compared with the LY293558 + CRM group. Sample size: *n* = 6 for each of the two groups. ** *p* < 0.01 (Student’s *t*-test, unequal variances).

Of the 119 rats that were exposed to soman, 22 had been implanted with subcutaneous electroencephalographic telemetry transmitters for EEG recordings. Three of these rats did not develop SE, and one died before treatment. Of the 18 remaining rats, 10 were treated with MDZ, of which 4 died during SE (before the 24 h), and 8 were treated with LY293558 + CRM, of which 2 died before the 24 h post-exposure period. Therefore, seizure measurements were obtained from 6 rats treated with MDZ and 6 rats treated with LY293558 + CRM. Significant difference between the two treatment groups was found only in the total duration of SE within 24 h post-exposure (Table 2 and Figure 2); the total duration includes all seizures that recur after the initial SE cessation by the anticonvulsant treatment, regardless of their duration.

### 3.2. EEG Power Integral and Body Temperature over 24 h After Soman Exposure

**Figure 3:**
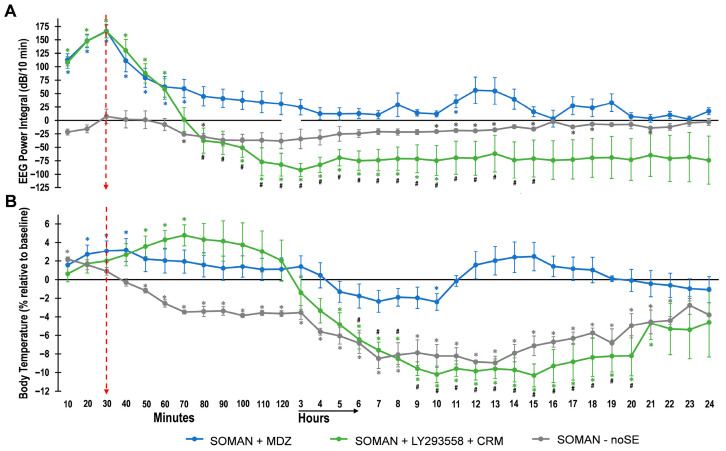
Changes in the EEG power integral and body temperature during the 24 h period after soman exposure in SE rats treated with MDZ or LY293558 + CRM, and in rats that did not develop SE (noSE). (**A**) EEG power data normalized to baseline (Power Integral = [10 × log(V^2^sample/V^2^ baseline)] × 10 min). (**B**) Corresponding changes in body temperature relative to baseline. Time zero corresponds to SE onset for the MDZ and LY293558 + CRM groups, and to 20 min post-exposure for the noSE group. The arrow at 30 min indicates the timepoint of anticonvulsant administration. In both (**A**,**B**), data are plotted in 10 min intervals for the first 2 h; each data point represents the mean ± standard error from 6 rats in each SE group and 3 rats in the noSE group. After the first 2 h, each data point represents the average over the 10 min preceding each full hour. Asterisks indicate significant differences from baseline (one-sample *t*-test for Power Integral; paired Student’s *t*-test for body temperature); number signs indicate significant differences between the two SE groups treated with MDZ or LY293558 + CRM (Student’s *t*-test). *^#^ *p* < 0.05.

To obtain a clearer view of the total power (energy) of the EEG signal during the 24 h post-exposure period, we calculated the power integral for SE rats treated with MDZ or LY293558 + CRM, as well as for noSE rats. Because exposure to OPs can disrupt thermoregulation [63], and both convulsive seizures and anticonvulsant treatments can affect body temperature—which in turn can influence seizures [64,65]—we plotted the EEG power integral together with body temperature to visualize changes in these two variables in parallel across the MDZ, LY293558 + CRM, and noSE groups (Figure 3).

The power integral rose sharply after SE onset (Figure 3A). Treatment with MDZ caused a rapid decline toward baseline, after which values remained near or slightly above baseline for the rest of the 24 h period. Body temperature in the MDZ group showed a small but significant increase before MDZ took effect, and then it remained slightly elevated, except for a transient reduction below baseline between 5 and 10 h post-exposure (Figure 3B).

Treatment with LY293558 + CRM produced a sharp decline of the power integral below baseline, and remained below baseline for the remaining 24 h period; during a substantial portion of this period, the reduction relative to baseline and the lower values compared with the MDZ group were statistically significant. Body temperature in the LY293558 + CRM group was above baseline for almost 2 h post-exposure, after which it dropped significantly and stayed low for the remaining observation period.

In the no-SE group, the power integral showed only a small decline below baseline, which was statistically significant at certain timepoints, as indicated in Figure 3A. Body temperature in this group was elevated at 30 min post-exposure, followed by a significant decline that persisted throughout the 24 h of recordings.

### 3.3. Further Analysis of the EEG in the MDZ, LY293558 + CRM, and noSE Groups—Increased Gamma Power in the noSE Rats

To better characterize EEG activity in SE rats treated with MDZ, those treated with LY293558 + CRM, as well as in noSE rats, we constructed spectrograms (frequency as a function of time) and analyzed the EEG signal using Fast Fourier Transform (FFT; power as a function of frequency) at different timepoints after soman exposure.

**Figure 4:**
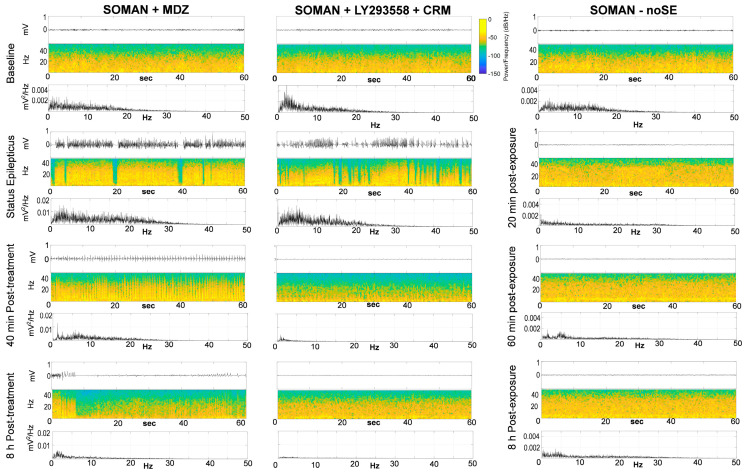
Spectrographic and Fast Fourier Transform (FFT) analyses of the EEG in representative rats from the MDZ, LY293558 + CRM, and noSE groups. Raw EEG traces (mV vs. time), spectrograms (Hz vs. time; color indicates power at each frequency in dB/Hz), and FFTs (power spectral density in mV^2^/Hz vs. frequency) are shown for representative rats that experienced SE after soman exposure and were treated with MDZ or LY293558 + CRM, as well as for rats who did not develop SE (noSE) despite exposure. Data are shown at baseline and at subsequent post-exposure timepoints, as indicated. EEG activity and spectrograms represent 60 s windows; FFTs indicate the power spectral density across frequencies within the same 60 s window. Note that the y-axis scale in some FFT plots changes to improve data visibility.

In the spectrograms of Figure 4, stripes of high energy in the low frequency range (yellow) persist at 40 min and 8 h after MDZ administration, whereas these frequencies have dissipated in the rat treated with LY293558 + CRM. In the noSE rat, the spectrograms are more scattered, but an energy increase in the gamma range (orange) is evident after soman exposure. The FFTs corroborate these findings: prominent peaks in the low to mid-frequency range persist at 40 min and 8 h after MDZ administration, whereas power across these frequencies is very low in the rat treated with LY293558 + CRM. In the noSE rat, power across frequencies remains comparable to baseline at all post-exposure timepoints.

**Figure 5:**
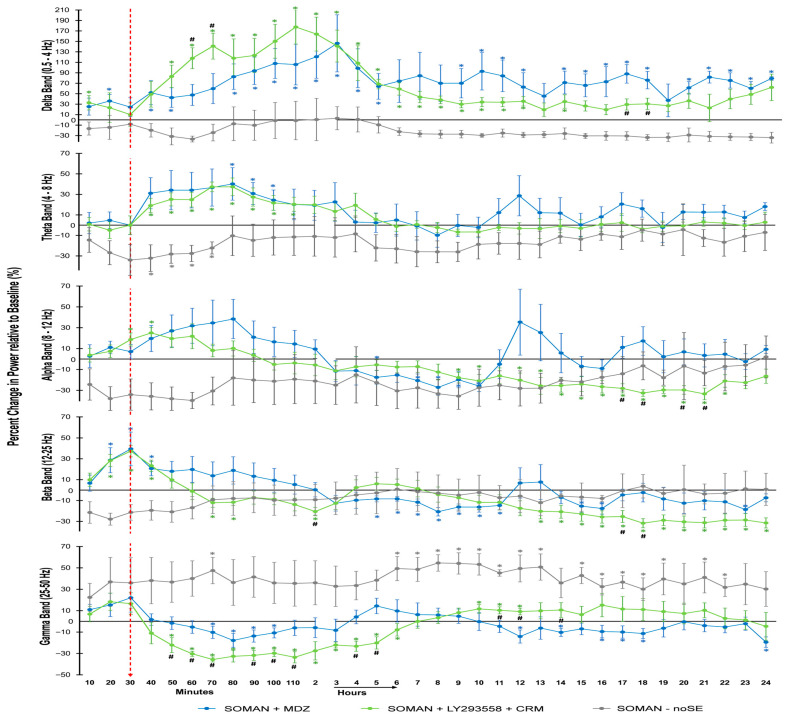
Changes in the distribution of EEG power across different frequency bands in SE rats treated with MDZ or LY293558 + CRM and in noSE rats. Time zero corresponds to SE onset for the MDZ and LY293558 + CRM groups, and to 20 min after soman exposure for the noSE group. Arrow at 30 min marks the timepoint of anticonvulsant administration. Power data are plotted in 10 min intervals for the first 2 h; each data point represents the mean ± standard error from 6 rats in each SE group and 3 rats in the noSE group. After the first 2 h, each data point represents the average over the 10 min preceding each full hour. Asterisks indicate significant differences from baseline (paired Student’s *t*-test); number signs indicate significant differences between the two SE groups treated with MDZ or LY293558 + CRM (Student’s *t*-test). *^#^ *p* < 0.05.

To gain insight into the distribution of EEG power across different frequency bands during SE and after MDZ or LY293558 + CRM treatment, as well as in soman-exposed rats that did not develop seizures, we performed a power spectral analysis of the 24 h EEG recordings from the MDZ, LY293558 + CRM, and noSE groups. The analysis was conducted for the delta (0.5–4 Hz), theta (4–8 Hz), alpha (8–12 Hz), beta (12–25 Hz), and gamma (25–50 Hz) bands (Figure 5). Data in Figure 5 are presented relative to baseline, with time zero corresponding to SE onset for the MDZ and LY293558 + CRM groups, and to 20 min after soman exposure for the noSE group.

Both the MDZ and the LY293558 + CRM groups displayed a significant increase in delta band power, which lasted for 14 h in the LY293558 + CRM group and throughout the 24 h of recording in the MDZ group. The increase was significantly higher in the LY293558 + CRM group compared with the MDZ group at 60–70 min after SE onset, and significantly higher in the MDZ group compared with the LY293558 + CRM group at 17–18 h. The noSE group did not show any significant changes in delta band power.

Theta band power was significantly elevated in the MDZ and LY293558 + CRM groups at many timepoints up to 110 min after SE onset; this was followed by a reduction, with values often above baseline in the MDZ group and slightly below baseline in the LY293558 + CRM group. There were no statistically significant differences between the two groups. In the noSE group, theta band power was reduced significantly from 40 to 70 min and remained slightly below baseline for the rest of the 24 h recording period.

The alpha band in the MDZ group showed wide fluctuations during the 24 h recordings, with a statistically significant reduction only between 7 and 10 h after SE onset. In the LY293558 + CRM group, alpha band power increased significantly between 30 and 40 min after SE onset, and subsequently decreased below baseline, significantly so at nearly all timepoints from 9 to 22 h. Between 17 and 21 h, alpha band power was significantly lower in the LY293558 + CRM group than in the MDZ group at most timepoints. In the noSE group, alpha band power remained below baseline, gradually approaching baseline during the 24 h of recording; however, the difference from baseline was not statistically significant at any timepoint.

Beta band power was significantly increased in both MDZ and LY293558 + CRM groups after SE onset, but started declining after anticonvulsant treatment. In the MDZ group, it was significantly below baseline between 5 and 11 and 15–16 h after SE onset. In the LY293558 + CRM group, the beta band power was significantly below baseline between 70 and 80 min, at 2 h, and from 13 h to the end of the recording period. At 2 h and between 17 and 18 h, beta power was significantly lower in the LY293558 + CRM group compared with the MDZ group. The noSE group did not exhibit any significant changes in beta band power.

Gamma-band power was significantly decreased in the MDZ group between 70 and 100 min and at several timepoints between 12 and 24 h after SE onset. In the LY293558 + CRM group, gamma power decreased significantly between 50 min and 6 h, but subsequently increased from 10 to 12 h. Between 50 and 100 min and again from 4 to 5 h, gamma power was significantly lower in the LY293558 + CRM group compared with the MDZ group, whereas at 11, 12, and 14 h it was higher in the LY293558 + CRM group. In the noSE group, gamma power was elevated above baseline throughout the 24 h of recordings, significantly so at 70 min and between 6 and 13 h and 15 and 22 h.

### 3.4. Protection 293558. + CRM, but Not MDZ, Against Neuronal Degeneration—Delayed Neurodegeneration in the noSE Group

**Figure 6:**
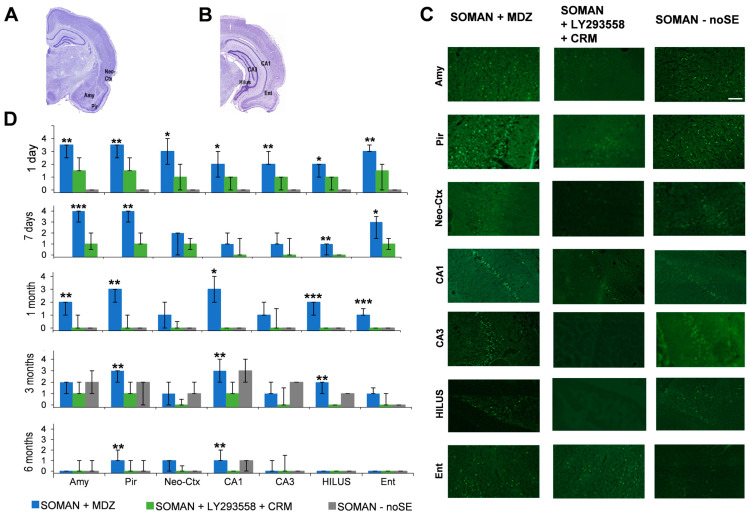
Neuronal degeneration after exposure of aged male rats to soman followed by treatment with MDZ or LY293558 + CRM—Delayed neurodegeneration in noSE rats. (**A**,**B**) Panoramic photomicrographs of Nissl-stained sections indicating the brain regions evaluated by Fluoro-Jade C staining. (**C**) Representative photomicrographs of FJC-stained sections from the brain regions where neurodegeneration was studied. The sections are from animals evaluated at 3 months after soman exposure. Total magnification is 100×. Scale bar is 50 μm. (**D**) Neuropathology scores (median and interquartile range) in the amygdala (Amy), piriform cortex (Pir), neocortical region (Neo-Ctx), hippocampal areas (CA1, CA3, and HILUS), and entorhinal cortex (Ent), at 1 day, 7 days, 1, 3, and 6 months post-exposure. Neurodegeneration in the noSE group was not assessed at 7 days. Sample sizes: MDZ, *n* = 5–6 rats per timepoint; LY293558 + CRM, *n* = 5–6 per timepoint; noSE = 5 rats per timepoint. Asterisks indicate significant differences between the two treatment groups. * *p* < 0.05, ** *p* < 0.01, *** *p* < 0.001 (Mann–Whitney U-test).

Neuronal degeneration was assessed in the amygdala, piriform cortex, temporal neocortex, CA1, CA3, and hilar areas of the hippocampus, as well as the entorhinal cortex (Figure 6A–D), at 1 day, 7 days, 1, 3, and 6 months after soman exposure. In the noSE group, neurodegeneration was not assessed at 7 days, since at both 1 day and 1 month there were no degenerating neurons in these rats. At each timepoint, the sample size was 5–6 rats in each of the two treatment groups and 5 rats in the noSE group.

At 1 day post-exposure, rats treated with LY293558 + CRM presented minimal to mild neurodegeneration (neurodegeneration scores in the amygdala: median = 1.5, IQR = 1.5~2.5; piriform cortex: median = 1.5, IQR = 1.5~2.5; temporal neocortex: median = 1, IQR = 0~2; CA1 area: median = 1, IQR = 0~1; CA3 area: median = 1, IQR = 0~1; hilus: median = 1, IQR = 0~1; entorhinal cortex: median = 1.5, IQR = 0~2), which was significantly less than the mild to severe neurodegeneration (depending on the brain region) in rats treated with MDZ (amygdala: median = 3.5, IQR = 2.5~3.5; piriform cortex: median = 3.5, IQR = 2.5~3.5; temporal neocortex: median = 3, IQR = 2~4; CA1: median = 2, IQR = 1~3; CA3: median = 2, IQR = 2~3; hilus: median = 2, IQR = 1~2; entorhinal cortex: median = 3, IQR = 3~3.5; Figure 6D). There were no degenerating neurons in the noSE rats at this post-exposure timepoint.

At 7 days post-exposure, neurodegeneration was again minimal to mild in rats treated with LY293558 + CRM, and mild to severe in MDZ-treated rats. The difference between the two groups was statistically significant in the amygdala (LY293558 + CRM: median = 1, IQR = 0.5~2; MDZ: median = 4, IQR = 3~4; *p* < 0.001), piriform cortex (LY293558 + CRM: median = 1, IQR = 1~2; MDZ: median = 4, IQR = 3~4; *p* < 0.01), the hilar region of the hippocampus (LY293558 + CRM: median = 0, IQR = 0~0; MDZ: median = 1, IQR = 0~1; *p* < 0.01), and the entorhinal cortex (LY293558 + CRM: median = 1, IQR = 0.5~1.5; MDZ: median = 3, IQR = 1.5~3.5; *p* < 0.05).

At 1 month post-exposure, neurodegeneration was virtually absent in rats treated with LY293558 + CRM, and mild to moderate in MDZ-treated rats. The difference between the two groups was statistically significant in the amygdala (LY293558 + CRM: median = 0, IQR = 0~1; MDZ: median = 2, IQR = 1~2; *p* < 0.01), piriform cortex (LY293558 + CRM: median = 0, IQR = 0~1; MDZ: median = 3, IQR = 2~3; *p* < 0.01), the CA1 hippocampal area (LY293558 + CRM: median = 0, IQR = 0~0; MDZ: median = 3, IQR = 2~4; *p* < 0.05), the hilar region (LY293558 + CRM: median = 0, IQR = 0~0; MDZ: median = 2, IQR = 1~2; *p* < 0.001), and the entorhinal cortex (LY293558 + CRM: median = 0, IQR = 0~0; MDZ: median = 1, IQR = 1~1.5; *p* < 0.001). There were still no degenerating neurons in the noSE rats at 1 month post-exposure.

At 3 months post-exposure, neurodegeneration was absent to mild in rats treated with LY293558 + CRM, and mild to moderate in MDZ-treated rats. The difference between the two groups was statistically significant in the piriform cortex (LY293558 + CRM: median = 1, IQR = 1~2; MDZ: median = 3, IQR = 2~3; *p* < 0.01), the CA1 hippocampal area (LY293558 + CRM: median = 1, IQR = 1~2; MDZ: median = 3, IQR = 3~4; *p* < 0.01), and the hilus (LY293558 + CRM: median = 0, IQR = 0~0; MDZ: median = 2, IQR = 1~2; *p* < 0.01). At this timepoint, mild to moderate neurodegeneration was also present in the noSE group (Figure 6D).

At 6 months post-exposure, neurodegeneration was virtually absent in rats treated with LY293558 + CRM, and only mild in some brain regions of the MDZ-treated group. The difference between the two groups was statistically significant in the piriform cortex (LY293558 + CRM: median = 0, IQR = 0~1; MDZ: median = 1, IQR = 1~2; *p* < 0.01) and the CA1 hippocampal area (LY293558 + CRM: median = 0, IQR = 0~0; MDZ: median = 1, IQR = 1~2; *p* < 0.01). Minimal to mild neurodegeneration was also present in the amygdala, the piriform cortex, and the CA1 hippocampal area of the noSE group (Figure 6D).

### 3.5. Protection by LY293558 + CRM, but Not MDZ, Against Neuronal and Interneuronal Loss—Delayed Neuronal and Interneuronal Loss in the BLA of the noSE Group

**Figure 7:**
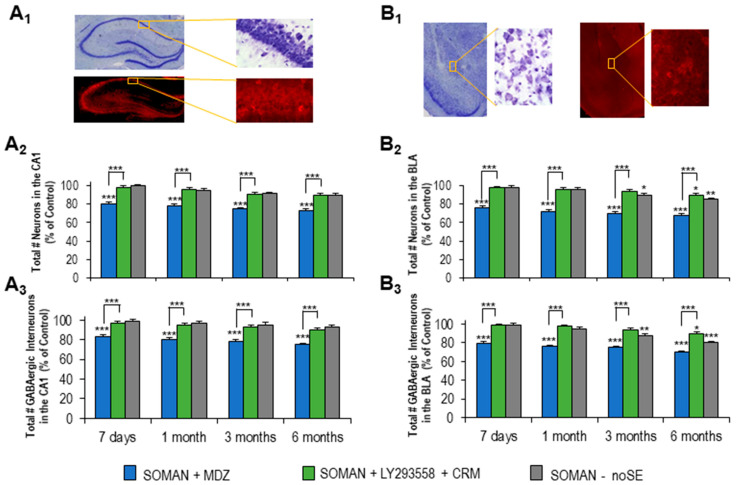
Νeuronal and interneuronal loss in the CA1 area and the BLA after exposure of aged male rats to soman followed by treatment with MDZ or LY293558 + CRM—Delayed loss in the BLA of noSE rats. (**A_1_**,**B_1_**) Photomicrographs of Nissl-stained and GAD-67–immunolabeled sections from the hippocampus (**A_1_**) and amygdala (**B_1_**). The rectangles mark the analyzed regions (CA1 and BLA, respectively); the corresponding magnified views are shown to the right of each section. (**A_2_**,**B_2_**) Group data of stereological estimation of the total number of neurons in the CA1 (**A_1_**) and BLA (**B_1_**) as % of the control group, at 7 days, 1, 3, and 6 months post-exposure. (**A_3_**,**B_3_**) Group data of stereological estimation of the total number of interneurons in the CA1 (**A_3_**) and BLA (**B_3_**) as % of the control group at the same post-exposure timepoints. Sample sizes for MDZ, LY293558 + CRM, and control groups: *n* = 5–6 rats for each group per timepoint; for the noSE group: *n* = 5 per timepoint. * *p* < 0.05, ** *p* < 0.01, and *** *p* < 0.001 (ANOVA, LSD post hoc test). Asterisks placed directly above a bar denote a significant difference from the control group.

Total neuronal and interneuronal loss was estimated in the CA1 hippocampal area (Figure 7A_1_) and the BLA (Figure 7B_1_) of SE rats treated with LY293558 + CRM or MDZ, as well as in noSE rats, at 7 days and at 1, 3, and 6 months after soman exposure. At each post-exposure timepoint, the sample size was 5–6 rats for each of the two treatment groups or the age-matched control group (not exposed to soman), and 5 rats for the noSE group.

In the CA1 area, the total number of neurons in the MDZ group at 7 days (477,971 ± 16,555), 1 month (452,186 ± 17,891), 3 months (440,237 ± 14,911), and 6 months (427,658 ± 15,453) post-exposure was significantly lower (*p* < 0.001) than that in the age-matched control group at the corresponding timepoints (1 week: 628,910 ± 16,550; 1 month: 630,182 ± 17,921; 3 months: 629,871 ± 14,989; 6 months: 625,314 ± 18,634) or in the LY293558 + CRM group (1 week: 613,983 ± 16,249; 1 month: 603,753 ± 15,920; 3 months: 601,995 ± 17,761; 6 months: 591,175 ± 29,981); the LY293558 + CRM group did not differ from the control at any post-exposure timepoint (Figure 7A_2_). Similarly, the number of interneurons in the CA1 area of the MDZ group at 7 days (52,125 ± 2871), 1 month (50,211 ± 3712), 3 months (49,485 ± 2917), and 6 months (46,187 ± 4187) post-exposure was significantly lower (*p* < 0.001) than that in the age-matched controls at the corresponding timepoints (1 week: 65,981 ± 1985; 1 month: 64,157 ± 2771; 3 months: 66,192 ± 5721; 6 months: 63,713 ± 3716) or in the LY293558 + CRM group (1 week: 65,387 ± 2091; 1 month: 62,331 ± 2788; 3 months: 62,220 ± 2353; 6 months: 59,890 ± 4761); the LY293558 + CRM group did not differ from the control at any post-exposure timepoint (Figure 7A_3_). There was no significant neuronal or interneuronal loss in the CA1 area of the noSE group at any post-exposure timepoint.

In the BLA, the total number of neurons in the MDZ group at 7 days (80,387 ± 3851), 1 month (76,100 ± 2871), 3 months (74,129 ± 3190), and 6 months (71,883 ± 2990) post-exposure was significantly lower (*p* < 0.001) than that in the age-matched control group at the corresponding timepoints (1 week: 105,773 ± 1760; 1 month: 103,161 ± 2580; 3 months: 104,912 ± 3121; 6 months: 102,998 ± 4129) or in the LY293558 + CRM group (1 week: 103,323 ± 2345; 1 month: 101,825 ± 3812; 3 months: 99,426 ± 3812; 6 months: 95,195 ± 3870); the total number of neurons in the BLA of the LY293558 + CRM group was significantly lower (*p* < 0.05) than that in the control group at 6 months post-exposure (Figure 7B_2_). Similarly, the number of interneurons in the BLA of the MDZ group at 7 days (10,262 ± 1887), 1 month (9886 ± 2912), 3 months (9743 ± 2166), and 6 months (9093 ± 2010) post-exposure was significantly lower (*p* < 0.001) than that in the age-matched control group at the corresponding timepoints (1 week: 12,991 ± 2018; 1 month: 13,812 ± 1989; 3 months: 12,713 ± 1659; 6 months: 11,995 ± 2871) or in the LY293558 + CRM group (1 week: 12,875 ± 1779; 1 month: 12,866 ± 2981; 3 months: 12,011 ± 3189; 6 months: 10,971 ± 3610); the total number of interneurons in the BLA of the LY293558 + CRM group was significantly lower (*p* < 0.05) than that in the control group at 6 months post-exposure (Figure 7B_3_).

In the noSE group, neuronal numbers in the BLA at 3 months (94,420 ± 1178) and 6 months (87,548 ± 2512) post-exposure were significantly lower than those in the control group (*p* < 0.05 and *p* < 0.01 for the 3 and 6 months, respectively; Figure 7B_2_). Interneuron numbers in the noSE group were also significantly lower compared with the control group at the same post-exposure timepoints (3 months: 11,080 ± 957, *p* < 0.01; and 6 months: 8776 ± 2287, *p* < 0.001; Figure 7B_3_).

### 3.6. Protection by LY293558 + CRM, but Not MDZ, Against Hippocampal and Amygdalar Atrophy—Delayed Amygdalar Atrophy in the noSE Group

**Figure 8:**
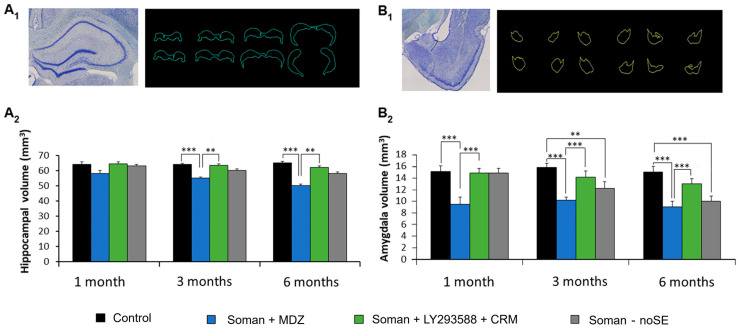
Hippocampal and amygdalar atrophy after exposure of aged male rats to soman followed by treatment with MDZ or LY293558 + CRM—Delayed reduction in amygdala volume in noSE rats. (**A_1_**,**B_1_**) Representative photomicrographs of Nissl-stained sections of the hippocampus (**A_1_**) and the amygdala (**B_1_**), with corresponding example tracings from the slice series used for volume estimation. (**A_2_**,**B_2_**) Group data showing the estimated volumes of the hippocampus (**A_2_**) and the amygdala (**B_2_**). Sample size *n* = 5 per group at each post-exposure timepoint. ** *p* < 0.01, *** *p* < 0.001 (one-Way ANOVA with Bonferroni post hoc).

The volumes of the hippocampus and the amygdala in SE rats treated with LY293558 + CRM or MDZ, as well as in noSE rats, were estimated at 1, 3, and 6 months after exposure to soman. Figure 8A_1_,B_1_ show examples of Nissl-stained sections from the hippocampus and the amygdala, respectively, from which tracings were made and subsequently used in Stereo Investigator 2017 to estimate volumes. Sample size at each of the evaluation timepoints was 5 rats per group.

There was no significant atrophy of the hippocampus in the LY293558 + CRM group at any post-exposure timepoint (Figure 8A_2_). In the MDZ-treated group, the volume of the hippocampus at 1 month post-exposure (58 ± 2 mm^3^) was not significantly different from that in the control group (64.2 ± 1.5 mm^3^; *p* = 0.1) or the LY293558 + CRM group (64.5 ± 1.4 mm^3^; *p* = 0.9). At 3 months post-exposure, hippocampal volume in the MDZ group (55 ± 0.9 mm^3^) was significantly smaller than that in the control (64 ± 0.8 mm^3^; *p* < 0.001) or the LY293558 + CRM group (63.4 ± 0.9 mm^3^; *p* < 0.01). Similarly, at 6 months post-exposure, hippocampal volume in the MDZ group (50 ± 1 mm^3^) was significantly smaller compared with the control (65 ± 0.9 mm^3^; *p* < 0.001) or the LY293558 + CRM group (62 ± 0.8 mm^3^; *p* < 0.01). There was no significant reduction in hippocampal volume of the noSE group at any post-exposure timepoint (Figure 8A_2_).

Amygdala volume was also not significantly reduced in the LY293558 + CRM group at any post-exposure timepoint (Figure 8B_2_). In the MDZ group, the volume of the amygdala at 1 month post-exposure (9.45 ± 1.05 mm^3^) was significantly smaller than that in the control group (15.1 ± 0.93 mm^3^; *p* < 0.001) or the LY293558 + CRM group (14.8 ± 1.23 mm^3^; *p* < 0.001). At 3 months post-exposure, amygdalar volume in the MDZ group (10.2 ± 0.35 mm^3^) was significantly smaller than that in the control (15.8 ± 0.76 mm^3^; *p* < 0.001) or the LY293558 + CRM group (14.1 ± 0.5 mm^3^; *p* < 0.001). Similarly, at 6 months post-exposure, amygdalar volume in the MDZ group (9 ± 0.35 mm^3^) was significantly smaller compared with the control (15 ± 1 mm^3^; *p* < 0.001) or the LY293558 + CRM group (13 ± 1 mm^3^; *p* < 0.01; Figure 8B_2_). In the noSE group, amygdala volume at 3 months (12.2 ± 1.1 mm^3^) and 6 months (10 ± 0.9 mm^3^) post-exposure was significantly smaller compared with the control group (*p* < 0.01 and *p* < 0.001 for the 3 and 6 months, respectively; Figure 8B_2_).

### 3.7. Protection by LY293558 + CRM, but Not MDZ, Against Reduction in Spontaneous IPSCs in the BLA—Delayed Reduction in Inhibition in the noSE Group

Principal neurons in the rat BLA display rhythmic “bursts” of summated sIPSCs, which are driven by rhythmic burst firing of GABAergic interneurons [61,62,66]. To determine whether the level of spontaneous inhibitory synaptic transmission in the BLA was altered after exposure to soman, we recorded sIPSCs from BLA principal neurons in in vitro brain slices obtained from SE rats treated with MDZ or LY293558 + CRM, as well as from noSE rats, at 1, 3, and 6 months post-exposure. We quantified this inhibitory activity by determining the percentage of principal neurons displaying sIPSC bursts, the total charge transferred by inhibitory currents within a 20 s time-window, and the burst frequency (number of sIPSC bursts within 10 s). The charge transferred was calculated as the sum of all inhibitory currents, irrespective of whether a neuron displayed sIPSC bursts.

**Figure 9:**
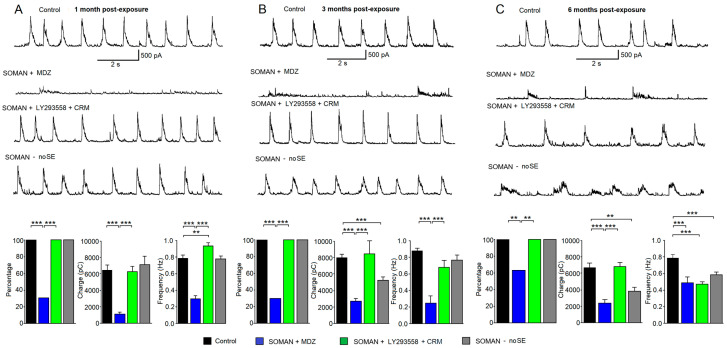
Reduction in spontaneous inhibitory activity in the BLA after exposure of aged male rats to soman followed by treatment with MDZ or LY293558 + CRM—Delayed impairment of inhibition in noSE rats. Traces in (**A**–**C**) are representative recordings of sIPSC “bursts” obtained from BLA principal neurons of a control rat (not exposed to soman), a soman-exposed rat treated with MDZ, a soman-exposed rat treated with LY293558 + CRM, and a soman-exposed rat that did not develop SE, at 1 (**A**), 3 (**B**), and 6 (**C**) months after soman exposure. Holding potential is +30 mV; there are no receptor antagonists present in the slice medium. Below the traces are group data corresponding to the three post-exposure timepoints, showing: percentage of neurons that displayed sIPSC bursts; total charge transferred by sIPSCs calculated during a 20 s time window for each recorded neuron; and frequency of sIPSC bursts calculated from those neurons that displayed sIPSC bursts. Sample size *n* ranges from 12 to 24 neurons. ** *p* < 0.001; *** *p* < 001 (Fisher exact test for the percentage of neurons displaying sIPSC bursts; Mann–Whitney test for the charge transferred and the frequency of sIPSC bursts).

At 1 month post-exposure, 100% of principal cells recorded from the BLA of the control—not exposed to soman—group (21 out of 21 neurons), the LY293558 + CRM group (19 out of 19 neurons), and the noSE group (15 out of 15 neurons) displayed sIPSC bursts; in contrast, 5 out of 16 recorded neurons displayed sIPSC bursts in the MDZ group (31%, significantly lower than the control and the LY293558 + CRM groups, *p* < 0.001; Figure 9A). The charge transferred by sIPSCs in the MDZ group was 1109 ± 240 pC (*n* = 16), which was significantly lower (*p* < 0.001) than in the control group (6477 ± 615 pC, *n* = 21) and the LY293558 + CRM group (6299 ± 648 pC, *n* = 24); neither the LY293558 + CRM group nor the noSE group (7184 ± 951 pC, *n* = 15) differed significantly from the control. The frequency of sIPSC bursts, when present in BLA neurons of the MDZ group, was 0.3 ± 0.04 Hz (*n* = 5), which was significantly lower (*p* < 0.001) than in the control group (0.79 ± 0.04, *n* = 21) and the LY293558 + CRM group (0.94 ± 0.04, *n* = 19); the frequency of sIPSC bursts was higher in the LY293558 + CRM group than in the control group (*p* < 0.01), whereas the burst frequency in the noSE group (0.78 ± 0.03, *n* = 15) did not differ from the control.

At 3 months post-exposure, again 100% of principal cells recorded from the BLA of the control group (12 out of 12 neurons), the LY293558 + CRM group (14 out of 14 neurons), and the noSE group (19 out of 19 neurons) displayed sIPSC bursts; in contrast, 6 out of 20 recorded neurons displayed sIPSC bursts in the MDZ group (30%; significantly lower than the control and the LY293558 + CRM groups, *p* < 0.001; Figure 9B). The charge transferred by sIPSCs in the MDZ group was 2691 ± 382 pC (*n* = 20), which was significantly lower (*p* < 0.001) than in the control group (8020 ± 435 pC, *n* = 12) and the LY293558 + CRM group (8508 ± 1591 pC, *n* = 14); the LY293558 + CRM group did not differ significantly from the control group, whereas the charge transferred in the noSE group (5267 ± 433 pC, *n* = 19) was significantly lower than the control (*p* < 0.001). The frequency of sIPSC bursts, when present in BLA neurons of the MDZ group, was 0.25 ± 0.09 Hz (*n* = 6), which was significantly lower (*p* < 0.001) than in the control group (0.88 ± 0.04, *n* = 12) or the LY293558 + CRM group (0.69 ± 0.08, *n* = 14); the burst frequency in neither the LY293558 + CRM group nor the noSE group (0.77 ± 0.06, *n* = 19) differed significantly from the control.

At 6 months post-exposure, 100% of principal cells recorded from the BLA of the control group (20 out of 20 neurons), the LY293558 + CRM group (17 out of 17 neurons), and the noSE group (13 out of 13 neurons) displayed sIPSC bursts; in contrast, 15 out of 24 recorded neurons displayed sIPSC bursts in the MDZ group (63%; significantly lower than the control and the LY293558 + CRM groups, *p* < 0.001; Figure 9C). The charge transferred by sIPSCs in the MDZ group was 2390 ± 418 pC (*n* = 24), which was significantly lower (*p* < 0.001) than in the control group (6718 ± 595 pC, *n* = 20) and the LY293558 + CRM group (6849 ± 513 pC, *n* = 17); the LY293558 + CRM group did not differ significantly from the control group, whereas the charge transferred in the noSE group (3858 ± 462 pC, *n* = 13) was significantly lower than the control (*p* < 0.01). The frequency of sIPSC bursts in both the MDZ group (0.49 ± 0.07 Hz, *n* = 15) and the LY293558 + CRM group (0.47 ± 0.03, *n* = 17) was significantly lower (*p* < 0.001) than in the control group (0.79 ± 0.04, *n* = 20); the burst frequency in the noSE group (0.59 ± 0.03) also differed significantly from the control (*p* < 0.001; Figure 9C).

## 4. Discussion

This study showed that, when aged rats are acutely exposed to 1.2 × LD_50_ of soman, a substantial percentage of them do not develop SE. Yet, despite the absence of SE, these rats displayed delayed neurodegeneration resulting in delayed but significant neuronal and interneuronal loss in the BLA, accompanied by reduction in amygdala volume. The rats that developed SE and were treated with MDZ exhibited extensive neurodegeneration, neuronal and interneuronal loss along with reduction in hippocampal and amygdalar volume, and impaired background inhibitory activity in the BLA, all of which were significant from early post-exposure timepoints. The SE rats treated with LY293558 + CRM had milder neurodegeneration that did not culminate in significant hippocampal or amygdalar atrophy. Preceding the neuropathology, the most noteworthy findings for the noSE rats during the 24 h post-exposure were a reduction in the EEG power integral—which was relatively small, albeit significant at several timepoints—a sustained increase in gamma power, and a significant drop in body temperature. In the SE rats, the MDZ group had a significantly longer duration of SE within the 24 h post-exposure period than the LY293558 + CRM group. The power integral was significantly increased in both experimental groups upon SE onset, started declining immediately after either anticonvulsant treatment, but remained above baseline in the MDZ group, while it decreased significantly below baseline in the group treated with LY293558 + CRM. Gamma-band power was significantly decreased below baseline in both SE groups in the early hours after either anticonvulsant treatment. In contrast, there was an increase in delta band power in both SE groups, lasting longer in the MDZ group. Body temperature dropped dramatically about 4 h after LY293558 + CRM treatment, whereas it remained relatively close to the baseline in the MDZ-treated group.

The difficulty we encountered in inducing SE in aged rats in the present study is not surprising, as we have previously observed lower susceptibility of aged rats to seizure initiation by soman exposure, together with higher mortality, compared with young-adult rats [43]. Similar findings have been reported in aged mice who had a higher seizure threshold—and higher mortality—in response to pentylenetetrazole injection [67]. This does not imply an overall lower seizure susceptibility in aged animals or humans. Age-related neurobiological changes and various comorbidities increase the risk of developing epilepsy in the elderly [68,69,70]. However, in response to certain seizure-inducing stimuli, such as chemoconvulsants or electroshock [71], the aged brain may exhibit a higher threshold for seizure generation, possibly due to age-related alterations in intrinsic neuronal properties, neurotransmitter systems, or mechanisms of seizure spread and propagation [72].

### 4.1. Mechanisms of Brain Damage in the noSE Group

It is widely accepted that long-term brain damage after acute OP exposure is caused primarily by the ensuing SE. This is plausible because prolonged SE of any etiology can cause brain injury [73,74,75], and the severity of neuropathology after acute OP exposure correlates with the severity and duration of SE [17,19,23]. However, other toxic mechanisms, independent of SE, may also contribute to brain injury after OP exposure [6,76,77]. Previous studies have shown that in the absence of SE, brain injury from acute OP exposure is not evident at early post-exposure timepoints [78], but appears as delayed damage [42]. The delayed appearance of neuropathology in the amygdala of the noSE group aligns with these earlier findings. Why damage in the noSE group was observed in the amygdala but not the hippocampus is unclear. However, it cannot be excluded that neuronal loss and atrophy would emerge also in the hippocampus at later post-exposure timepoints (beyond 6 months).

Could seizure activity have contributed to neuronal damage in the amygdala of the noSE group? The EEG power integral indicated that the overall level of electrical activity in this group was weak and significantly below baseline at several timepoints, suggesting no large-scale hyperexcitability during the first 24 h after soman exposure. However, this does not preclude localized, low-amplitude seizure activity in deep limbic structures that would not be detectable with our cortical telemetry. In addition, progressively developing hyperexcitability in the BLA during the course of 6 months cannot be excluded; the delayed reduction in spontaneous inhibitory activity suggests that hyperexcitability could have become significant at 6 months post-exposure.

Acetylcholinesterase activity is inhibited after acute soman exposure even in rats that do not develop SE (though significantly less in the BLA) [78]. The resulting increase in acetylcholine might damage neurons by increasing intracellular Ca^++^ via over-activation of cholinergic receptors. Sustained increases in intracellular Ca^++^ have been observed after low-dose, repeated OP exposures, suggesting that they can occur without SE [79].

Other mechanisms of OP-induced, SE-independent delayed neuropathology may involve neuroinflammation, oxidative stress, and disruption of axonal transport [76,80]. Some of these mechanisms may also be independent of acetylcholinesterase inhibition [81,82]; for example, expression of inflammatory proteins was increased by exposure to OP insecticides in cell cultures of primary human astrocytes [83].

### 4.2. Severe Brain Damage After Treatment of SE with MDZ; Protection by LY293558 + CRM

From the rats that developed SE, those treated with MDZ displayed severe hippocampal and amygdalar pathology and impaired background inhibition in the BLA, suggesting hyperexcitability [84] with implications for development of epilepsy and chronically increased anxiety [35]. In contrast, SE rats given LY293558 + CRM showed reduced neuronal and interneuronal counts only in the BLA at 6 months, but this was not sufficient to result in significant amygdalar atrophy, at least by the 6-month post-exposure timepoint. The neuroprotection provided by the antiglutamatergic treatment likely reflected the significantly shorter total duration of SE compared with the MDZ group, as determined by the raw EEG data and 24 h power-integral calculations. While the total EEG power in the MDZ group remained above baseline throughout the 23.5 h after treatment, in the LY293558 + CRM group it dropped significantly below baseline by 50 min post-treatment, suggesting a deeper level of suppression and reduced excitability capable of dampening residual epileptiform or pro-epileptic activity. By strongly suppressing electrical activity, LY293558 + CRM may reduce the excitotoxic activity that occurs during seizures and the downstream cascades that lead to neuronal death and neurodegeneration. As noted in the introduction and thoroughly discussed in previous reports [27,35], the failure of MDZ to reduce electrical activity sufficiently to prevent hyperexcitability and seizure reocurrence is likely due to internalization of GABA_A_ receptors during SE and other mechanisms that weaken synaptic inhibition.

### 4.3. Do Changes in Body Temperature Play a Role in Brain Damage?

Exposure to OPs can cause hypothermia in both humans [63] and animals [85,86,87] during the early hours post-exposure; the mechanisms may involve primarily central effects [88,89,90] secondary to overstimulation of cholinergic receptors following inhibition of acetylcholinesterase. Consistent with this effect of OPs, a significant decrease in body temperature occurred in the noSE group, beginning about 70 min after soman injection. This temperature drop may have played a role in maintaining the EEG power integral below baseline and in limiting neuropathology, since hypothermia suppresses seizure activity [64] and can have neuroprotective effects [91].

Body temperature in the SE rats followed a different time course from that in the noSE group; it began to rise after SE onset in both treatment groups, consistent with the known effect of seizures on body temperature [65]. After MDZ treatment, body temperature returned close to baseline and remained there for the 24 h post-exposure period. In contrast, after treatment with LY293558 + CRM, body temperature continued to increase for another ~40 min before a marked reduction that persisted throughout the recordings. In both SE groups, the decrease in EEG total power after anticonvulsant administration preceded the fall in body temperature. Treatment with LY293558 + CRM permitted a decline in body temperature (and may have contributed to it, since some anticonvulsants have hypothermic effects [92,93,94]) to levels and with a time course similar to those in the noSE group; in contrast, in the MDZ group, the sustained elevated electrical activity may have played a role in keeping body temperature near baseline. The marked body temperature decrease in the LY293558 + CRM group for about 20 h may have enhanced neuroprotection by lowering metabolic demand and stabilizing neuronal function (membrane properties, ion-pump activity, and excessive release of excitatory neurotransmitters).

### 4.4. Do Changes in the Power of Specific Frequencies Have Predictive Value for the Occurrence of SE or Neuropathology Outcomes?

Spectral analysis of the EEG for 24 h after soman exposure revealed a sustained increase in gamma-band power in the noSE group. Slow gamma (25–50 Hz, as measured here) is generated by inhibitory neurons acting as pacemakers, reflects the level of synchronous activity in neuronal networks, and is associated with active cognitive states during information processing [95,96]. Evidence suggests a link between increases in gamma power and reduction in seizure activity. In a case study, Le Van Quyen et al. [97] reported that an increase in gamma power was accompanied by a decrease in spike frequency in an epileptic patient. In animals, Testylier et al. [98] found that increased gamma power after acute soman exposure occurred only in rats that did not develop SE. They proposed that, during OP poisoning, rats that are resilient to induction of SE mount a strong emergency response with heightened arousal and increased gamma activity, which prevents the synchronization of neuronal firing that characterizes epileptic activity [98]. More recent work shows that reduced gamma activity is linked to network hypersynchrony and the generation of epileptiform discharges in both animals [99,100] and humans [101]. Indeed, gamma entrainment has been suggested as a potential therapeutic approach for a range of neurological and neuropsychiatric disorders [96,102]. Thus, the sustained gamma elevation observed in noSE animals could reflect compensatory excitatory–inhibitory rebalancing and preserved or enhanced interneuron synchrony, which may represent a resilience trait that helps prevent seizure initiation.

In the SE groups, gamma-band power was reduced after anticonvulsant treatment, significantly more in the LY293558 + CRM than in the MDZ group. This reduction in gamma power may reflect the anesthetic effect of the anticonvulsants and the lower level of consciousness; the difference between the two treatments in the magnitude of the reduction may reflect the different mechanisms by which these treatments act and affect the balance of excitation and inhibition on which gamma oscillations depend [103]. At 9 h post-exposure, gamma-band power in the LY293558 + CRM group returned and rose above baseline, whereas in the MDZ group it dropped below baseline; this earlier rebound may indicate a more rapid restoration of normal cortical network activity and functional connectivity, consistent with the limited neuropathology observed in this group.

The severity of neuropathology after OP exposure has been particularly associated with increases in delta-band power. In nerve agent-exposed rats experiencing untreated SE, delta-band power remains markedly elevated for about 45 h, gradually returning to baseline within the next 1 to 4 days [38,104,105]. It has been observed that the level of increase at about 24 h post-exposure correlates with the severity of neuropathology [104]. In the present study, both SE groups displayed significantly increased delta power. However, while delta power in the LY293558 + CRM group was no longer significantly above baseline 15 h after SE onset, it remained significantly above baseline in the MDZ group for the remainder of the 24 h recording period. Considering that the MDZ group displayed severe neuropathology while the LY293558 + CRM group showed only limited damage, the early delta elevation in the latter group could be due mainly to a deep level of sedation—consistent with the concomitant reduction in gamma power—rather than an indication of neuronal exhaustion and damage.

## 5. Conclusions

The present study supports previous evidence [42] that even a single, acute OP exposure can cause brain damage—albeit delayed and relatively mild—without induction of SE; the delayed appearance of neuropathology offers an opportunity for intervention to prevent it or minimize it. A rise in gamma power in rats that do not develop SE after acute exposure to soman, first reported in 1999 [98] and confirmed in the present study, supports the view that gamma-band power can have predictive value for seizure occurrence. The pronounced and prolonged reduction in body temperature experienced by both groups that showed only limited and delayed brain damage (the noSE group and the LY293558 + CRM group), but not by the MDZ group, which developed severe neuropathology, suggests that a substantial drop in body temperature after OP exposure may be predictive of milder neuropathology outcomes. Lastly, as demonstrated in the present study, the antiglutamatergic therapy for soman-induced SE consisting of LY293558 and CRM is far more neuroprotective than MDZ in aged rats, as previously shown also in immature and young-adult male and female rats [23,35]. As soman is generally considered the most potent of the better-known nerve agents (partly due to the rapid aging of the soman–acetylcholinesterase complex [5,6,7]) the results are likely to apply to other nerve agents and organophosphates; however, it is unknown whether they also apply to A-series nerve agents [5], since less is known about the comparative severity of SE and the resulting brain damage induced by these compounds. The present findings are likely to be relevant to SE of other etiologies, and suggest that antagonizing glutamate receptors can circumvent the consequences of the GABA_A_ receptor-trafficking cascade that is largely responsible for the failure of benzodiazepines to effectively terminate SE, particularly when administered with some delay.

## Figures and Tables

**Table 2:** Seizure metrics—based on EEG recordings—in aged male rats exposed to soman and treated with MDZ or LY293558 + CRM: latency to cessation of the initial SE after administration of MDZ or LY293558 + CRM, duration of the initial SE (up to its transient cessation by anticonvulsant treatment), and total duration of SE within 24 h post-exposure.

Groups	Latency to Cessation of Initial SE	Duration of Initial SE	Total Duration of SE (in 24 h)
MDZ	202.8 ± 66	223.3 ± 58	553.8 ± 99
LY293558 + CRM	52.6 ± 12 *p =* 0.07	90.4 ± 13 *p* = 0.074	91.2 ± 13 ** p = 0.005

Data are minutes (mean ± standard error). Sample size *n* = 6 for each treatment group. ** *p* < 0.01 (*t*-test, unequal variances).

## Data Availability

The data supporting the findings are available upon request.

## References

[1] Mauricio E.A., Freeman W.D. (2011). Status epilepticus in the elderly: Differential diagnosis and treatment. Neuropsychiatr. Dis. Treat..

[2] Sadeghi M., Eshraghi M., Akers K.G., Hadidchi S., Kakara M., Nasseri M., Mahulikar A., Marawar R. (2020). Outcomes of status epilepticus and their predictors in the elderly—A systematic review. Seizure.

[3] Woodward M.R., Armahizer M.J., Wang T.I., Badjatia N., Johnson E.L., Gilmore E.J. (2025). Status epilepticus in older adults: A critical review. Epilepsia.

[4] Newmyer L., Verdery A.M., Wang H., Margolis R. (2022). Population aging, demographic metabolism, and the rising tide of late middle age to older adult loneliness around the world. Popul. Dev. Rev..

[5] Aroniadou-Anderjaska V., Apland J.P., Figueiredo T.H., de Araujo Furtado M., Braga M.F. (2020). Acetylcholinesterase inhibitors (nerve agents) as weapons of mass destruction: History, mechanisms of action, and medical countermeasures. Neuropharmacology.

[6] Aroniadou-Anderjaska V., Figueiredo T.H., de Araujo Furtado M., Pidoplichko V.I., Braga M.F.M. (2023). Mechanisms of organophosphate toxicity and the role of acetylcholinesterase inhibition. Toxics.

[7] Sirin G.S., Zhang Y. (2014). How is acetylcholinesterase phosphonylated by soman? An ab initio QM/MM molecular dynamics study. J. Phys. Chem. A.

[8] Olsen R.W. (2018). GABA_A_ receptor: Positive and negative allosteric modulators. Neuropharmacology.

[9] Newmark J. (2019). Therapy for acute nerve agent poisoning: An update. Neurol. Clin. Pract..

[10] Gorecki L., Pejchal J., Torruellas C., Korabecny J., Soukup O. (2024). Midazolam—A diazepam replacement for the management of nerve agent-induced seizures. Neuropharmacology.

[11] Reddy S.D., Reddy D.S. (2015). Midazolam as an anticonvulsant antidote for organophosphate intoxication—A pharmacotherapeutic appraisal. Epilepsia.

[12] Shih T., McDonough J.H., Koplovitz I. (1999). Anticonvulsants for soman-induced seizure activity. J. Biomed. Sci..

[13] Jones D.M., Esmaeil N., Maren S., MacDonald R.L. (2002). Characterization of pharmacoresistance to benzodiazepines in the rat Li-pilocarpine model of status epilepticus. Epilepsy Res..

[14] Goodkin H.P., Liu X., Holmes G.L. (2003). Diazepam terminates brief but not prolonged seizures in young, naïve rats. Epilepsia.

[15] Gilat E., Kadar T., Levy A., Rabinovitz I., Cohen G., Kapon Y., Sahar R., Brandeis R. (2005). Anticonvulsant treatment of sarin-induced seizures with nasal midazolam: An electrographic, behavioral, and histological study in freely moving rats. Toxicol. Appl. Pharmacol..

[16] Wu X., Kuruba R., Reddy D.S. (2018). Midazolam-resistant seizures and brain injury after acute intoxication of diisopropylfluorophosphate, an organophosphate pesticide and surrogate for nerve agents. J. Pharmacol. Exp. Ther..

[17] Apland J.P., Aroniadou-Anderjaska V., Figueiredo T.H., Rossetti F., Miller S.L., Braga M.F. (2014). The limitations of diazepam as a treatment for nerve agent-induced seizures and neuropathology in rats: Comparison with UBP302. J. Pharmacol. Exp. Ther..

[18] de Araujo Furtado M., Aroniadou-Anderjaska V., Figueiredo T.H., Apland J.P., Braga M.F.M. (2020). Electroencephalographic analysis in soman-exposed 21-day-old rats and the effects of midazolam or LY293558 with caramiphen. Ann. N.Y. Acad. Sci..

[19] Figueiredo T.H., Aroniadou-Anderjaska V., Pidoplichko V.I., Apland J.P., Braga M.F.M. (2022). Antiseizure and neuroprotective efficacy of midazolam in comparison with tezampanel (LY293558) against soman-induced status epilepticus. Toxics.

[20] Lumley L., Miller D., Muse W.T., Marrero-Rosado B., de Araujo Furtado M., Stone M., McGuire J., Whalley C. (2019). Neurosteroid and benzodiazepine combination therapy reduces status epilepticus and long-term effects of whole-body sarin exposure in rats. Epilepsia Open.

[21] Supasai S., González E.A., Rowland D.J., Hobson B., Bruun D.A., Guignet M.A., Soares S., Singh V., Wulff H., Saito N. (2020). Acute administration of diazepam or midazolam minimally alters long-term neuropathological effects in the rat brain following acute intoxication with diisopropylfluorophosphate. Eur. J. Pharmacol..

[22] Gage M., Rao N.S., Samidurai M., Putra M., Vasanthi S.S., Meyer C., Wang C., Thippeswamy T. (2022). Soman (GD) rat model to mimic civilian exposure to nerve agent: Mortality, video-EEG based status epilepticus severity, sex differences, spontaneously recurring seizures, and brain pathology. Front. Cell. Neurosci..

[23] de Araujo Furtado M., Aroniadou-Anderjaska V., Figueiredo T.H., Pidoplichko V.I., Apland J.P., Rossetti K., Braga M.F.M. (2024). Preventing long-term brain damage by nerve agent-induced status epilepticus in rat models applicable to infants: Significant neuroprotection by tezampanel combined with caramiphen but not by midazolam treatment. J. Pharmacol. Exp. Ther..

[24] Naylor D.E., Liu H., Niquet J., Wasterlain C.G. (2013). Rapid surface accumulation of NMDA receptors increases glutamatergic excitation during status epilepticus. Neurobiol. Dis..

[25] Goodkin H.P., Joshi S., Mtchedlishvili Z., Brar J., Kapur J. (2008). Subunit-specific trafficking of GABA(A) receptors during status epilepticus. J. Neurosci..

[26] Deeb T.Z., Nakamura Y., Frost G.D., Davies P.A., Moss S.J. (2013). Disrupted Cl (−) homeostasis contributes to reductions in the inhibitory efficacy of diazepam during hyperexcited states. Eur. J. Neurosci..

[27] Aroniadou-Anderjaska V., Figueiredo T.H., de Araujo Furtado M., Pidoplichko V.I., Lumley L.A., Braga M.F.M. (2024). Alterations in GABAA receptor-mediated inhibition triggered by status epilepticus and their role in epileptogenesis and increased anxiety. Neurobiol. Dis..

[28] Hill C.E., Parikh A.O., Ellis C., Myers J.S., Litt B. (2017). Timing is everything: Where status epilepticus treatment fails. Ann. Neurol..

[29] Ullal G., Fahnestock M., Racine R. (2005). Time-dependent effect of kainate-induced seizures on glutamate receptor GluR5, GluR6, and GluR7 mRNA and protein expression in rat hippocampus. Epilepsia.

[30] Rajasekaran K., Todorovic M., Kapur J. (2012). Calcium-permeable AMPA receptors are expressed in a rodent model of status epilepticus. Ann. Neurol..

[31] Joshi S., Rajasekaran K., Sun H., Williamson J., Kapur J. (2017). Enhanced AMPA receptor-mediated neurotransmission on CA1 pyramidal neurons during status epilepticus. Neurobiol. Dis..

[32] Amakhin D.V., Soboleva E.B., Ergina J.L., Malkin S.L., Chizhov A.V., Zaitsev A.V. (2018). Seizure-induced potentiation of AMPA receptor-mediated synaptic transmission in the entorhinal cortex. Front. Cell. Neurosci..

[33] Bleakman R., Schoepp D.D., Ballyk B., Bufton H., Sharpe E.F., Thomas K., Ornstein P.L., Kamboj R.K. (1996). Pharmacological discrimination of GluR5 and GluR6 kainate receptor subtypes by (3S,4aR,6R,8aR)-6-[2-(1(2)H-tetrazole-5-yl) ethyl]decahydroisoquinoline-3-carboxylic acid. Mol. Pharmacol..

[34] Apland J.P., Aroniadou-Anderjaska V., Figueiredo T.H., Pidoplichko V.I., Rossetti K., Braga M.F.M. (2018). Comparing the antiseizure and neuroprotective efficacy of LY293558, diazepam, caramiphen, and LY293558-caramiphen combination against soman in a rat model relevant to the pediatric population. J. Pharmacol. Exp. Ther..

[35] Figueiredo T.H., Aroniadou-Anderjaska V., Pidoplichko V.I., Furtado M.A., Rossetti K., Lumley L.A., Braga M.F.M. (2025). Sex-dependent differences in the antiseizure and neuroprotective effects of midazolam after soman exposure: Superior, sex-independent efficacy of tezampanel and caramiphen. Exp. Neurol..

[36] Raveh L., Chapman S., Cohen G., Alkalay D., Gilat E., Rabinovitz I., Weissman B.A. (1999). The involvement of the NMDA receptor complex in the protective effect of anticholinergic drugs against soman poisoning. Neurotoxicology.

[37] Figueiredo T.H., Aroniadou-Anderjaska V., Qashu F., Apland J.P., Pidoplichko V., Stevens D., Ferrara T.M., Braga M.F. (2011). Neuroprotective efficacy of caramiphen against soman and mechanisms of its action. Br. J. Pharmacol..

[38] Apland J.P., Aroniadou-Anderjaska V., Figueiredo T.H., de Araujo Furtado M., Braga M.F.M. (2018). Full protection against soman-induced seizures and brain damage by LY293558 and caramiphen combination treatment in adult rats. Neurotox. Res..

[39] Poza J.J. (2007). Management of epilepsy in the elderly. Neuropsychiatr. Dis. Treat..

[40] Siniscalchi A. (2010). Treatment of epilepsy in the elderly people. BMC Geriatr..

[41] Seo J.G., Cho Y.W., Kim K.T., Kim D.W., Yang K.I., Lee S.T., Byun J.I., No Y.J., Kang K.W., Kim D. (2020). Pharmacological treatment of epilepsy in elderly patients. J. Clin. Neurol..

[42] González E.A., Rindy A.C., Guignet M.A., Calsbeek J.J., Bruun D.A., Dhir A., Andrew P., Saito N., Rowland D.J., Harvey D.J. (2020). The chemical convulsant diisopropylfluorophosphate (DFP) causes persistent neuropathology in adult male rats independent of seizure activity. Arch. Toxicol..

[43] Apland J.P., Aroniadou-Anderjaska V., Figueiredo T.H., Prager E.M., Olsen C.H., Braga M.F.M. (2017). Susceptibility to soman toxicity and efficacy of LY293558 against soman-induced seizures and neuropathology in 10-month-old male rats. Neurotox. Res..

[44] Racine R.J. (1972). Modification of seizure activity by electrical stimulation. II. Motor seizure. Electroencephalogr. Clin. Neurophysiol..

[45] Figueiredo T.H., Qashu F., Apland J.P., Aroniadou-Anderjaska V., Souza A.P., Braga M.F. (2011). The GluK1 (GluR5) kainate/α-amino-3-hydroxy-5-methyl-4-isoxazolepropionic acid receptor antagonist LY293558 reduces soman-induced seizures and neuropathology. J. Pharmacol. Exp. Ther..

[46] Lumley L.A., Nguyen D.A., de Araujo Furtado M., Niquet J., Linz E.O., Schultz C.R., Stone M.F., Wasterlain C.G. (2024). Efficacy of lacosamide and rufinamide as adjuncts to midazolam–ketamine treatment against cholinergic-induced status epilepticus in rats. J. Pharmacol. Exp. Ther..

[47] de Araujo Furtado M., Zheng A., Sedigh-Sarvestani M., Lumley L., Lichtenstein S., Yourick D. (2009). Analyzing large data sets acquired through telemetry from rats exposed to organophosphorous compounds: An EEG study. J. Neurosci. Methods.

[48] Lumley L.A., Rossetti F., de Araujo Furtado M., Marrero-Rosado B., Schultz C.R., Schultz M.K., Niquet J., Wasterlain C.G. (2019). Dataset of EEG power integral, spontaneous recurrent seizure and behavioral responses following combination drug therapy in soman-exposed rats. Data Brief.

[49] Apland J.P., Figueiredo T.H., Qashu F., Aroniadou-Anderjaska V., Souza A.P., Braga M.F. (2010). Higher susceptibility of the ventral versus the dorsal hippocampus and the posteroventral versus anterodorsal amygdala to soman-induced neuropathology. Neurotoxicology.

[50] Aroniadou-Anderjaska V., Fritsch B., Qashu F., Braga M.F. (2008). Pathology and pathophysiology of the amygdala in epileptogenesis and epilepsy. Epilepsy Res..

[51] Choy M., Dadgar-Kiani E., Cron G.O., Duffy B.A., Schmid F., Edelman B.J., Asaad M., Chan R.W., Vahdat S., Lee J.H. (2022). Repeated hippocampal seizures lead to brain-wide reorganization of circuits and seizure propagation pathways. Neuron.

[52] Fastenrath M., Coynel D., Spalek K., Milnik A., Gschwind L., Roozendaal B., Papassotiropoulos A., de Quervain D.J. (2014). Dynamic modulation of amygdala-hippocampal connectivity by emotional arousal. J. Neurosci..

[53] Šimić G., Tkalčić M., Vukić V., Mulc D., Španić E., Šagud M., Olucha-Bordonau F.E., Vukšić M., Hof P.R. (2021). Understanding emotions: Origins and roles of the amygdala. Biomolecules.

[54] Qasim S.E., Mohan U.R., Stein J.M., Jacobs J. (2023). Neuronal activity in the human amygdala and hippocampus enhances emotional memory encoding. Nat. Hum. Behav..

[55] Gundersen H.J., Jensen E.B., Kiêu K., Nielsen J. (1999). The efficiency of systematic sampling in stereology—Reconsidered. J. Microsc..

[56] Schmitz C., Hof P.R. (2000). Recommendations for straightforward and rigorous methods of counting neurons based on a computer simulation approach. J. Chem. Neuroanat..

[57] Gundersen H.J., Bendtsen T.F., Korbo L., Marcussen N., Møller A., Nielsen K., Nyengaard J.R., Pakkenberg B., Sørensen F.B., Vesterby A. (1988). Some new, simple and efficient stereological methods and their use in pathological research and diagnosis. APMIS.

[58] Paxinos G., Watson C. (2005). The Rat Brain in Stereotaxic Coordinates.

[59] Sah P., Faber E.S., Lopez de Armentia M., Power J. (2003). The amygdaloid complex: Anatomy and physiology. Physiol. Rev..

[60] Pidoplichko V.I., Aroniadou-Anderjaska V., Prager E.M., Figueiredo T.H., Almeida-Suhett C.P., Miller S.L., Braga M.F. (2014). ASIC1a activation enhances inhibition in the basolateral amygdala and reduces anxiety. J. Neurosci..

[61] Pidoplichko V.I., Aroniadou-Anderjaska V., Figueiredo T.H., Wilbraham C., Braga M.F.M. (2021). Increased inhibitory activity in the basolateral amygdala and decreased anxiety during estrus: A potential role for ASIC1a channels. Brain Res..

[62] Aroniadou-Anderjaska V., Pidoplichko V.I., Figueiredo T.H., Braga M.F.M. (2018). Oscillatory synchronous inhibition in the basolateral amygdala and its primary dependence on NR2A-containing NMDA receptors. Neuroscience.

[63] Moffatt A., Mohammed F., Eddleston M., Azher S., Eyer P., Buckley N.A. (2010). Hypothermia and fever after organophosphorus poisoning in humans—A prospective case series. J. Med. Toxicol..

[64] Niquet J., Baldwin R., Gezalian M., Wasterlain C.G. (2015). Deep hypothermia for the treatment of refractory status epilepticus. Epilepsy Behav..

[65] Pollandt S., Bleck T.P. (2018). Thermoregulation in epilepsy. Handb. Clin. Neurol..

[66] Popescu A.T., Paré D. (2011). Synaptic interactions underlying synchronized inhibition in the basal amygdala: Evidence for existence of two types of projection cells. J. Neurophysiol..

[67] Nokubo M., Kitani K., Ohta M., Kanai S., Sato Y., Masuda Y. (1986). Age-dependent increase in the threshold for pentylenetetrazole-induced maximal seizure in mice. Life Sci..

[68] Stephen L.J., Brodie M.J. (2000). Epilepsy in elderly people. Lancet.

[69] Liu S., Yu W., Lü Y. (2016). The causes of new-onset epilepsy and seizures in the elderly. Neuropsychiatr. Dis. Treat..

[70] Sen A., Jette N., Husain M., Sander J.W. (2020). Epilepsy in older people. Lancet.

[71] Kitani K., Sato Y., Kanai S., Nokubo M., Ohta M., Masuda Y. (1985). Age related increased threshold for electroshock seizure in BDF1 mice. Life Sci..

[72] Lee J., Kim H.J. (2022). Normal aging induces changes in the brain and neurodegeneration progress: Review of the structural, biochemical, metabolic, cellular, and molecular changes. Front. Aging Neurosci..

[73] Fujikawa D.G. (2005). Prolonged seizures and cellular injury: Understanding the connection. Epilepsy Behav..

[74] Gaínza-Lein M., Barcia Aguilar C., Piantino J., Chapman K.E., Sánchez Fernández I., Amengual-Gual M., Anderson A., Appavu B., Arya R., Brenton J.N. (2021). Pediatric Status Epilepticus Research Group. Factors associated with long-term outcomes in pediatric refractory status epilepticus. Epilepsia.

[75] Bosque Varela P., Machegger L., Steinbacher J., Oellerer A., Pfaff J., McCoy M., Trinka E., Kuchukhidze G. (2024). Brain damage caused by status epilepticus: A prospective MRI study. Epilepsy Behav..

[76] Naughton S.X., Terry A.V. (2018). Neurotoxicity in acute and repeated organophosphate exposure. Toxicology.

[77] Tsai Y.H., Lein P.J. (2021). Mechanisms of organophosphate neurotoxicity. Curr. Opin. Toxicol..

[78] Prager E.M., Aroniadou-Anderjaska V., Almeida-Suhett C.P., Figueiredo T.H., Apland J.P., Braga M.F. (2013). Acetylcholinesterase inhibition in the basolateral amygdala plays a key role in the induction of status epilepticus after soman exposure. Neurotoxicology.

[79] Phillips K.F., Santos E., Blair R.E., Deshpande L.S. (2019). Targeting intracellular calcium stores alleviates neurological morbidities in a DFP-based rat model of Gulf War illness. Toxicol. Sci..

[80] Banks C.N., Lein P.J. (2012). A review of experimental evidence linking neurotoxic organophosphorus compounds and inflammation. Neurotoxicology.

[81] Terry A.V. (2012). Functional consequences of repeated organophosphate exposure: Potential non-cholinergic mechanisms. Pharmacol. Ther..

[82] Lorke D.E., Oz M. (2025). A review on oxidative stress in organophosphate-induced neurotoxicity. Int. J. Biochem. Cell Biol..

[83] Mense S.M., Sengupta A., Lan C., Zhou M., Bentsman G., Volsky D.J., Whyatt R.M., Perera F.P., Zhang L. (2006). The common insecticides cyfluthrin and chlorpyrifos alter the expression of a subset of genes with diverse functions in primary human astrocytes. Toxicol. Sci..

[84] Prager E.M., Pidoplichko V.I., Aroniadou-Anderjaska V., Apland J.P., Braga M.F. (2014). Pathophysiological mechanisms underlying increased anxiety after soman exposure: Reduced GABAergic inhibition in the basolateral amygdala. Neurotoxicology.

[85] Timofeeva O.A., Gordon C.J. (2001). Changes in EEG power spectra and behavioral states in rats exposed to the acetylcholinesterase inhibitor chlorpyrifos and muscarinic agonist oxotremorine. Brain Res..

[86] Nguyen D.A., Niquet J., Marrero-Rosado B., Schultz C.R., Stone M.F., de Araujo Furtado M., Biney A.K., Lumley L.A. (2025). Age differences in organophosphorus nerve agent-induced seizure, blood–brain barrier integrity, and neurodegeneration in midazolam-treated rats. Exp. Neurol..

[87] Steier H.G., Schultz C.R., Niquet J., Nguyen D.A., Stone M.F., Biney A.K., de Araujo Furtado M., Wasterlain C.G., Lumley L.A. (2025). Perampanel as a second-line therapy to midazolam reduces soman-induced status epilepticus and neurodegeneration in rats. Epilepsia Open.

[88] Unal C.B., Demiral Y., Ulus I.H. (1998). The effects of choline on body temperature in conscious rats. Eur. J. Pharmacol..

[89] Gordon C.J., Grantham T.A. (1999). Effect of central and peripheral cholinergic antagonists on chlorpyrifos-induced changes in body temperature in the rat. Toxicology.

[90] Takahashi A., Ishimaru H., Ikarashi Y., Kishi E., Maruyama Y. (2001). Hypothalamic cholinergic regulation of body temperature and water intake in rats. Auton. Neurosci..

[91] Wang Y., Liu P.P., Li L.Y., Zhang H.M., Li T. (2011). Hypothermia reduces brain edema, spontaneous recurrent seizure attack, and learning memory deficits in the kainic acid treated rats. CNS Neurosci. Ther..

[92] Lin M.T., Chen C.F., Pang I.H. (1978). Effect of ketamine on thermoregulation in rats. Can. J. Physiol. Pharmacol..

[93] Clark S.M., Lipton J.M. (1981). Effects of diazepam on body temperature of the aged squirrel monkey. Brain Res. Bull..

[94] Dowden J., Reid C., Dooley P., Corbett D. (1999). Diazepam-induced neuroprotection: Dissociating the effects of hypothermia following global ischemia. Brain Res..

[95] Buzsáki G., Wang X.J. (2012). Mechanisms of gamma oscillations. Annu. Rev. Neurosci..

[96] Ichim A.M., Barzan H., Moca V.V., Nagy-Dabacan A., Ciuparu A., Hapca A., Vervaeke K., Muresan R.C. (2024). The gamma rhythm as a guardian of brain health. Elife.

[97] Le Van Quyen M., Adam C., Lachaux J.P., Martinerie J., Baulac M., Renault B., Varela F.J. (1997). Temporal patterns in human epileptic activity are modulated by perceptual discriminations. Neuroreport.

[98] Testylier G., Tonduli L., Lallement G. (1999). Implication of gamma band in soman-induced seizures. Acta Biotheor..

[99] Verret L., Mann E.O., Hang G.B., Barth A.M., Cobos I., Ho K., Devidze N., Masliah E., Kreitzer A.C., Mody I. (2012). Inhibitory interneuron deficit links altered network activity and cognitive dysfunction in Alzheimer model. Cell.

[100] Martinez-Losa M., Tracy T.E., Ma K., Verret L., Clemente-Perez A., Khan A.S., Cobos I., Ho K., Gan L., Mucke L. (2018). Nav1.1-overexpressing interneuron transplants restore brain rhythms and cognition in a mouse model of Alzheimer’s disease. Neuron.

[101] Matsumoto J.Y., Stead M., Kucewicz M.T., Matsumoto A.J., Peters P.A., Brinkmann B.H., Danstrom J.C., Goerss S.J., Marsh W.R., Meyer F.B. (2013). Network oscillations modulate interictal epileptiform spike rate during human memory. Brain.

[102] Guan A., Wang S., Huang A., Qiu C., Li Y., Li X., Wang J., Wang Q., Deng B. (2022). The role of gamma oscillations in central nervous system diseases: Mechanism and treatment. Front. Cell Neurosci..

[103] Atallah B.V., Scanziani M. (2009). Instantaneous modulation of gamma oscillation frequency by balancing excitation with inhibition. Neuron.

[104] McDonough J.H., Clark T.R., Slone T.W., Zoeffel D., Brown K., Kim S., Smith C.D. (1998). Neural lesions in the rat and their relationship to EEG delta activity following seizures induced by the nerve agent soman. Neurotoxicology.

[105] Carpentier P., Foquin A., Dorandeu F., Lallement G. (2001). Delta activity as an early indicator for soman-induced brain damage: A review. Neurotoxicology.

